# Supplementary material for: Patient-derived xenografts and organoids model therapy response in prostate cancer
Source: Nat Commun. 2021 Feb 18;12:1117. doi: 10.1038/s41467-021-21300-6 (PMC7892572; doi:10.1038/s41467-021-21300-6)
Supplement: Supplementary file 3 — Description of Additional Supplementary Files [file 41467_2021_21300_MOESM3_ESM.docx]

**Description of Supplementary Files**

**File Name: Supplementary Data 1**

**Description:** List of somatic mutations identified by ion torrent sequencing in the PNPCa PDX List of somatic mutations identified by ion torrent sequencing in intact, castrated, replaced castrated+testosterone) and prolonged castrated+ testosterone PNPCa PDX tumors. Gene name, chromosome position, type of mutation and variant allele fraction are indicated.

**File Name: Supplementary Data 2**

**Description:** List of all somatic mutations identified by WES in the PNPCa, LAPC9, BM18 PDXs and organoids List of somatic mutations, single nucleotide variants and insertion-deletions identified by WES after Disambiquate analysis in the PDXs and PDX-derived organoids from all models; PNPCa, LAPC9, BM18.

**File Name: Supplementary Data 3**

**Description:** List of Nexus FDA approved compound library used for the organoid screens The following information are included; drug name, catalog number (Sellchekchem), Molecular Weight (MW), Chemical Abstracts Service identifier (CAS Number), indication of area of clinical use, molecular target description, chemical structure information (SMILES) and stock concentration.
