# Supplementary material for: Patient-derived xenografts and organoids model therapy response in prostate cancer
Source: Nat Commun. 2021 Feb 18;12:1117. doi: 10.1038/s41467-021-21300-6 (PMC7892572; doi:10.1038/s41467-021-21300-6)
Supplement: Supplementary file 1 — Supplementary Information [file 41467_2021_21300_MOESM1_ESM.pdf]

Supplementary Information (Supplementary Figures)

Patient-derived xenografts and organoids model therapy response in prostate cancer

Sofia Karkampouna* and Federico La Manna*, et al.

Supp. Fig. 1

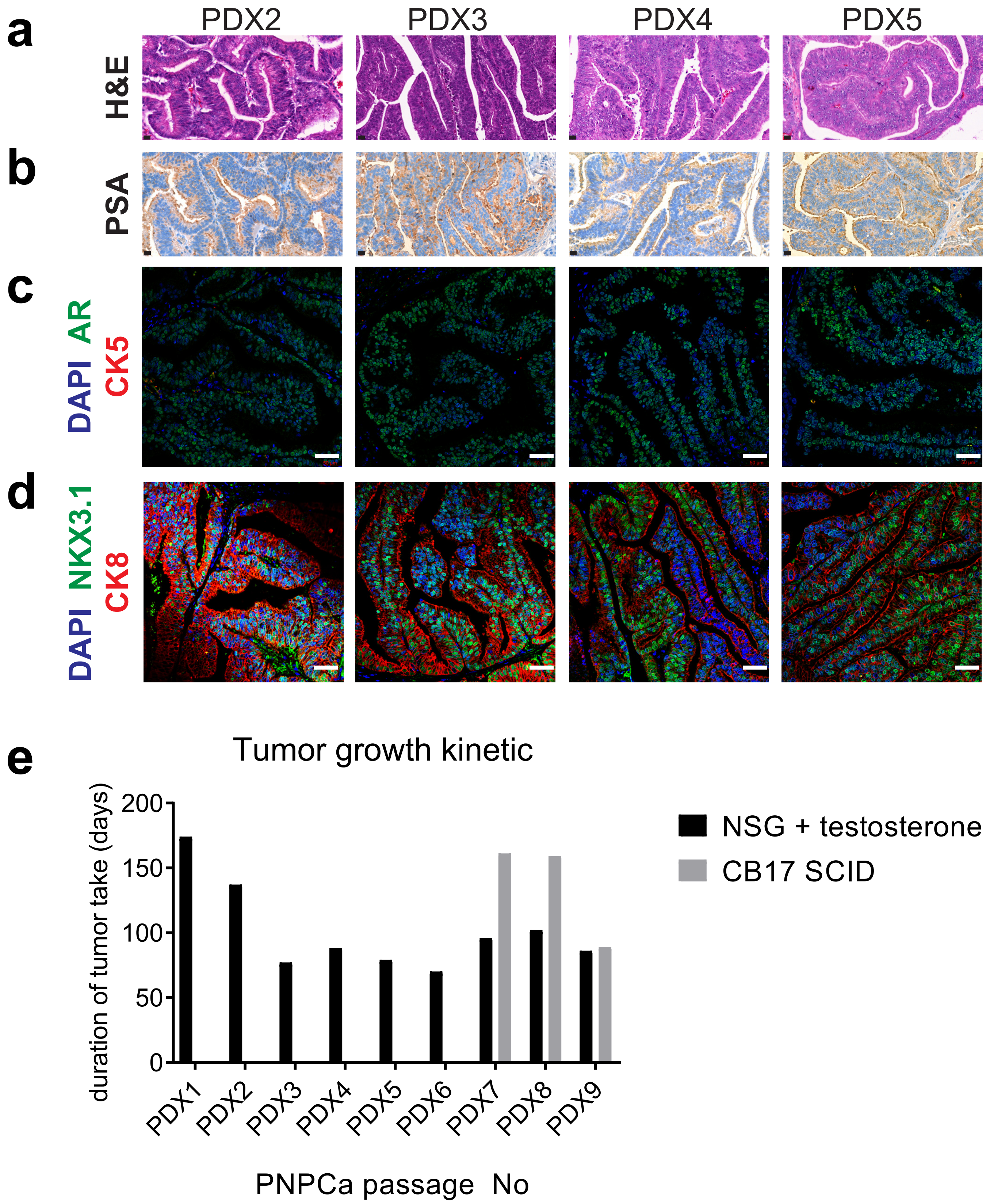

Supp.Figure 1. Maintenance of luminal epithelial morphology of the PDX and tumor growth kinetic in different genetic backgrounds, related to Fig.1

a. Histological morphology of PNPCa PDX passages (PDX2-PDX5) as assessed by Hematoxylin and Eosin staining (H&E). Scale bars 20μm. **b.** PSA protein expression. Scale bars 20μm. **c.** Expression of AR (green), CK5 (red) assessed by immunofluorescence, DAPI (blue) marks the nuclei. Scale bars 50μm. **d.** Expression of NKX3.1 (green), CK8 (red) assessed by immunofluorescence. Scale bars 50μm. **e.** Duration of tumor take (days between subcutaneous implantation and tumor growth of ~1cm^3 in immunocompromised strains NOD-*scid* IL2Rgamma^{null} (NSG). Testosterone supplementation was performed weekly to ensure grafting success with weekly testosterone injections. Starting from passage 7, the PDX tumor engrafted in less immunocompromised strain CB17 SCID with testosterone supplementation. Each bar represents data from one animal.

Supp. Fig. 2

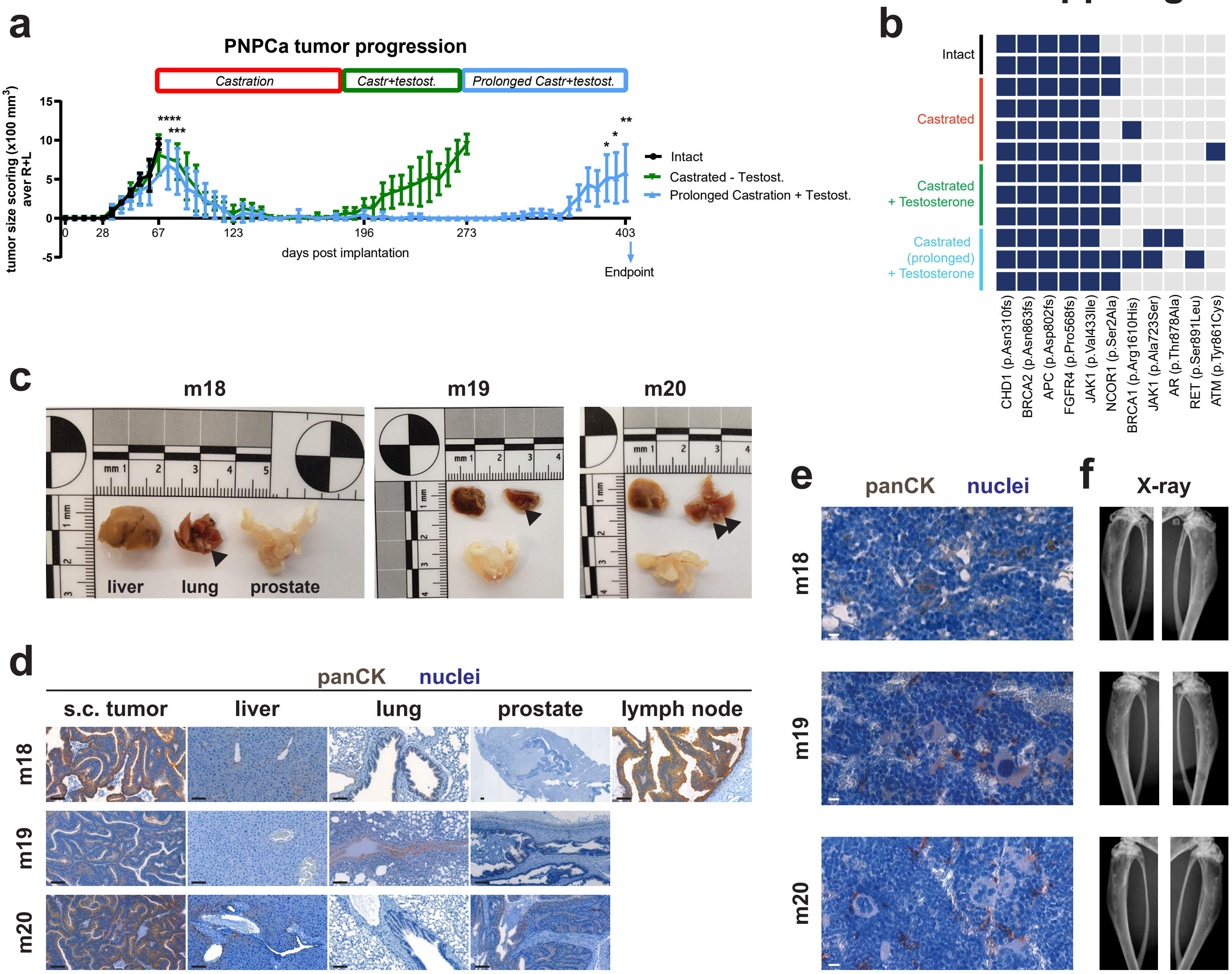

Supp.Figure 2. Effects of prolonged castration, regeneration and micrometastasis detection in PNPCa PDX, related to Fig.1

a. PDX tumor growth progression in time. Groups; 1.Intact tumors (black line; N=2), 2. Castration followed by testosterone re-administration (Castr+Testost, green line; N=4, N=3 from day 203-252, N=2 from day 252-273), 3. Prolonged castration followed by testosterone re-administration (Prolonged Castr+Testost, blue line; N=3, castration at day 67, testosterone from day 273 to 403). Sample size (N) represents biologically independent animals, each engrafted with two tumors. Center of line represents arithmetic mean, error bars represent SD. Ordinary one-way ANOVA compared to day 0: day 68 (****) p<0.0001, day 74 (***) p=0.001, day 392 and 399 (*) p=0.0161, day 403 (**) p=0.0018. Genomic analysis on PCa panel by Ion Torrent sequencing on FFPE samples of PNPCa tumors from intact, castrated, castrated+testosterone and prolonged castr+testosterone, at endpoints. **c.** Liver, lung and prostate tissues from NSG mice with subcutaneous PNPCa tumors at endpoint (N=3; Castr+Testost group, mouse id reported above each image). The follow-up of castration was a total of 40 weeks. No spontaneous regrowth of tumor was observed, until supplemented with testosterone for seven weeks. **d.** Human pan-cytokeratins (panCK) staining on subcutaneous PNPCa tumor, liver, lung, prostate (anterior lobe), lymph node from mice reported in (c). Scale bars, 100 μm. **e.** Representative human panCK staining (scale bars, 20 μm) and **f.** X-ray images of left and right tibias of mice reported in (c).

Supp. Fig. 3

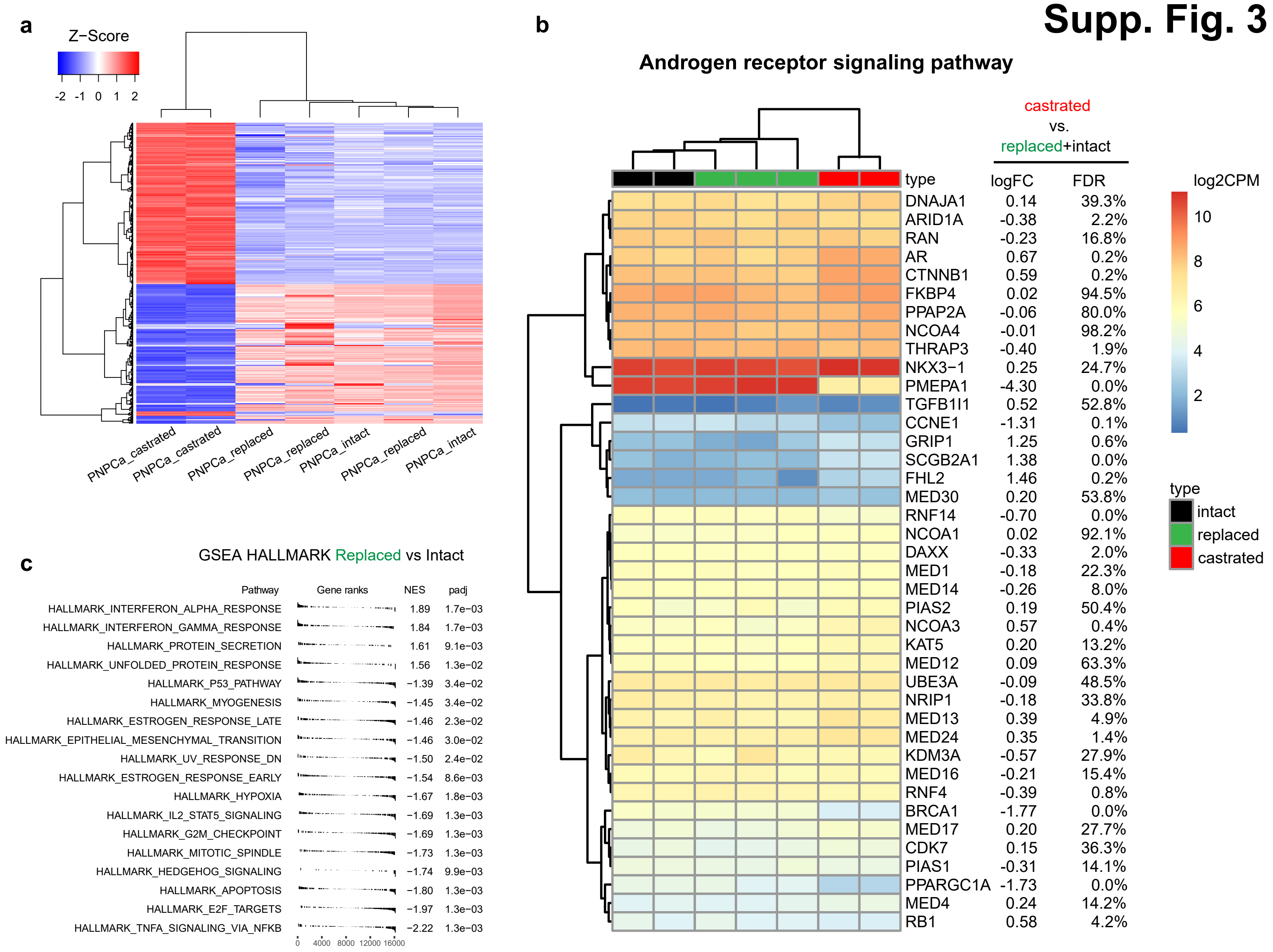

Supp.Figure 3. RNAseq confirms testosterone re-administration following castration reverses the transcriptomic changes of castration, and is similar to an intact tumor, related to Fig.1

a. Heatmap of non-hierarchical clustering of the top 500 variable genes across all samples indicated (N=3 for Replaced group, N=2 for all other treatment groups). **b.** Heatmap of expression of AR pathway genes and differential gene expression values for Castrated versus pooled Intact and Replaced groups. Differential expression was inferred using the quasi-likelihood F-test based on a negative binomial dispersion model of raw counts and a design matrix (edgeR package). **c.** Gene set enrichment analysis plot of statistically significant enrichment of Hallmark pathways based on the differential expression analysis of the Replaced versus the Intact group (adjusted p-value < 0.05). Differential expression was inferred using the quasi-likelihood F-test based on a negative binomial dispersion model of raw counts and a design matrix (edgeR package). Gene set enrichment scores were inferred using the standard GSEA method (correlation-weighted Kolmogorov–Smirnov test, the normalized ES, and the false discovery rate calculation).

Supp. Fig. 4

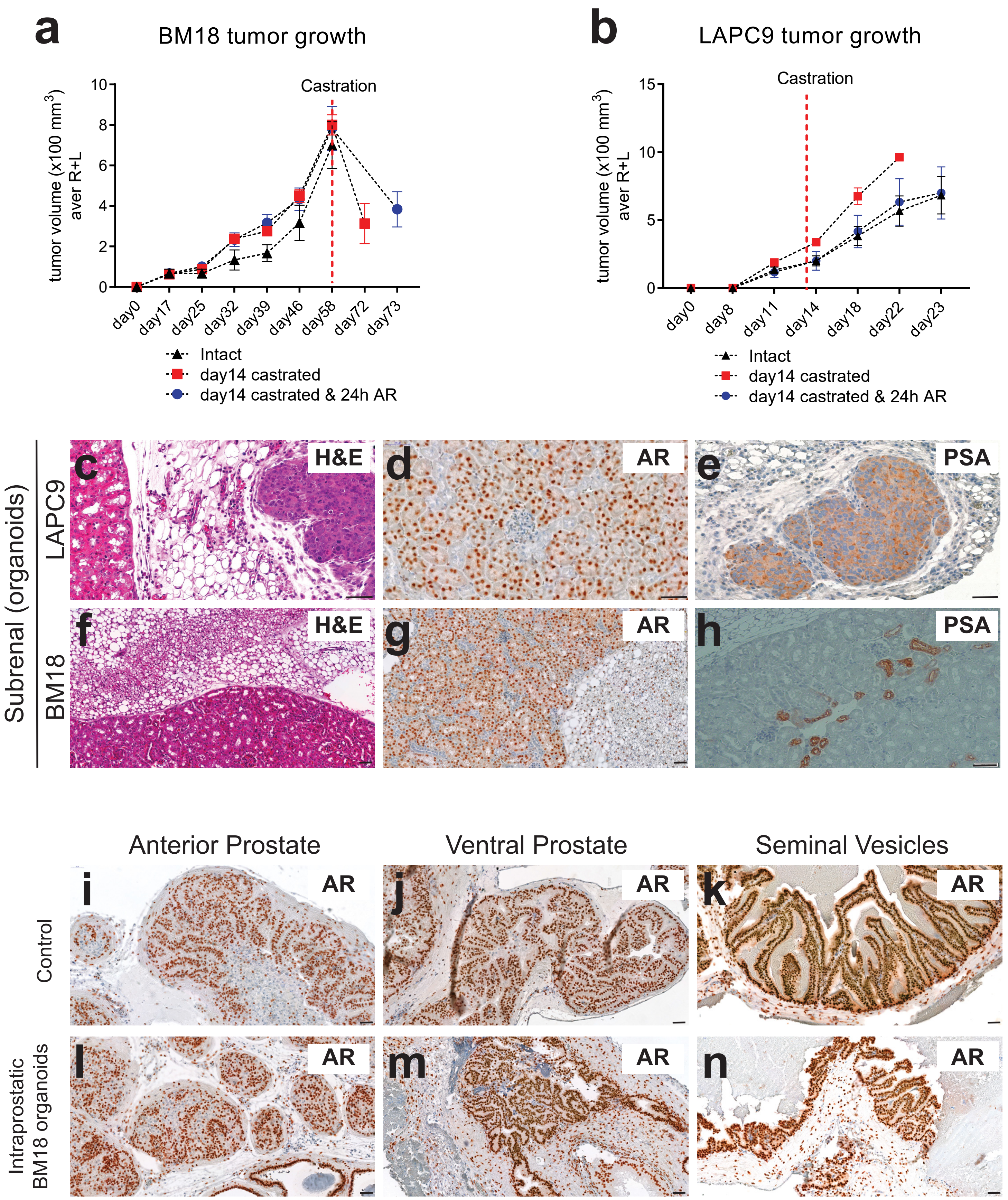

Supp.Figure 4. *In vivo* tumorigenicity of BM18 and LAPC9 organoids, related to Fig. 2

a,b. Tumor growth kinetic of BM18 (a) and LAPC9 (b) subcutaneous PDX tumors in response to castration and testosterone (DHT). Mean values ± SD are reported. Values refer to independent tumor samples, from 3-4 engrafted animals per condition. For intact and 24h AR groups, N = 6; for castrated groups N = 8. **c-h.** Subrenal implantation of LAPC9 organoids **(c-e)** and of BM18 organoids **(f-h)**; H&E staining (c,f), AR staining (d,g) and PSA staining (e,h) of the subrenal tumor growth. **i-n.** AR staining of control prostatic tissues (Anterior, Ventral Prostate and Seminal Vesicles) **(i-k)** and prostatic tissues from intraprostatic inoculation of BM18 organoids in the anterior prostate **(l-n)**. Scale bars 50μm.

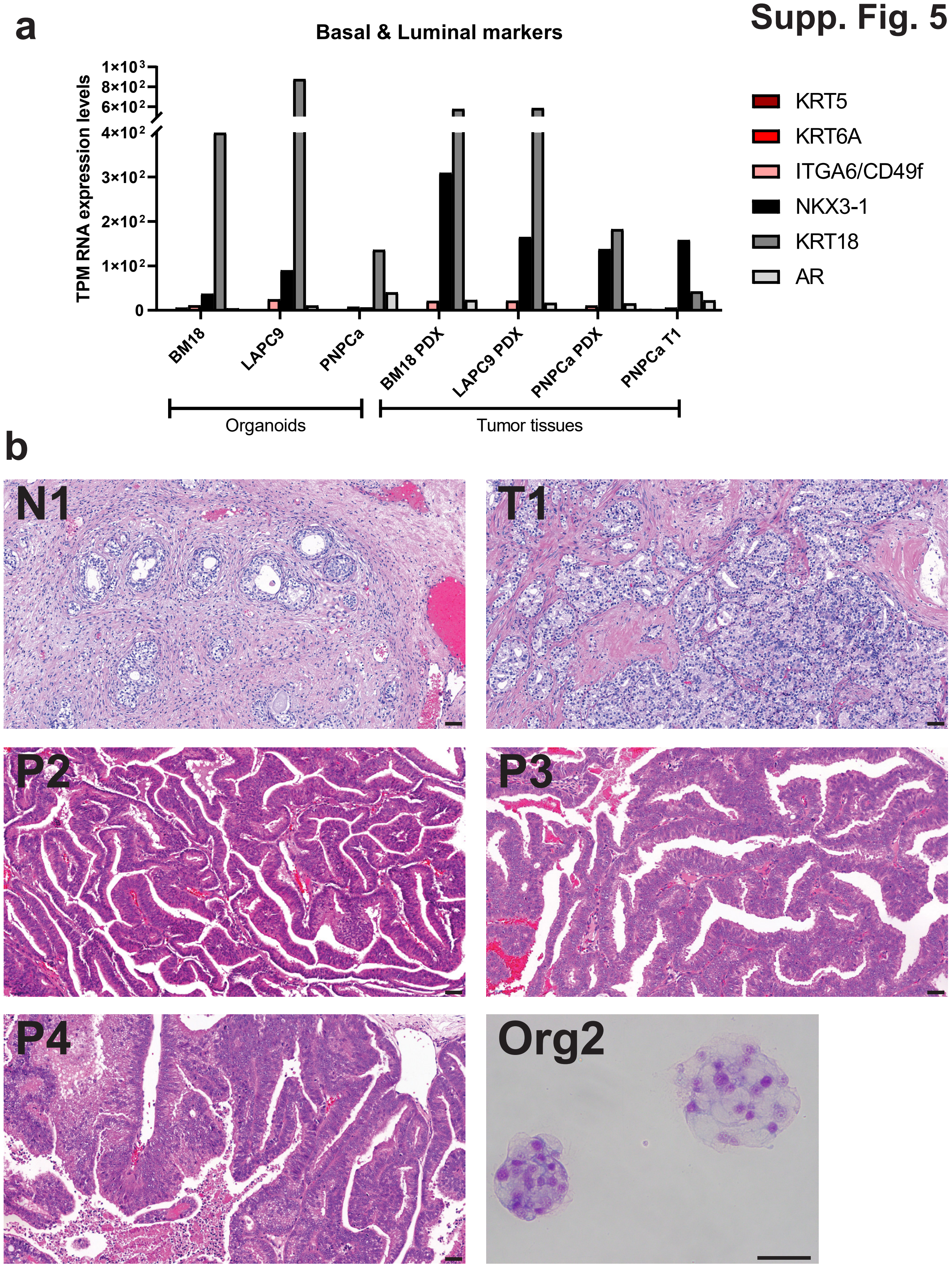

Supp.Figure 5. Expression of basal and luminal markers and histological morphology of PNPCa PDX and organoids, related to Fig.2

a. Gene expression levels for basal (*ITGA6/CD49f, KRT5, KRT6*) and luminal (*NKX3.1, AR, KRT18*) markers in different PDXs and PDX-derived organoids. Data reported as transcripts-per-million (TPM) from RNA sequencing experiments, N = 1 each. **b.** Hematoxylin and Eosin staining of the samples used for exome sequencing; patient-derived material (primary tumor TUR-P ("T1") and non-carcinoma control "N1"), PDX passages from PNPCa met (p2, p3, p4) and PDX-derived organoids (Org2). Scale bars 50µm.

Supp. Fig. 6

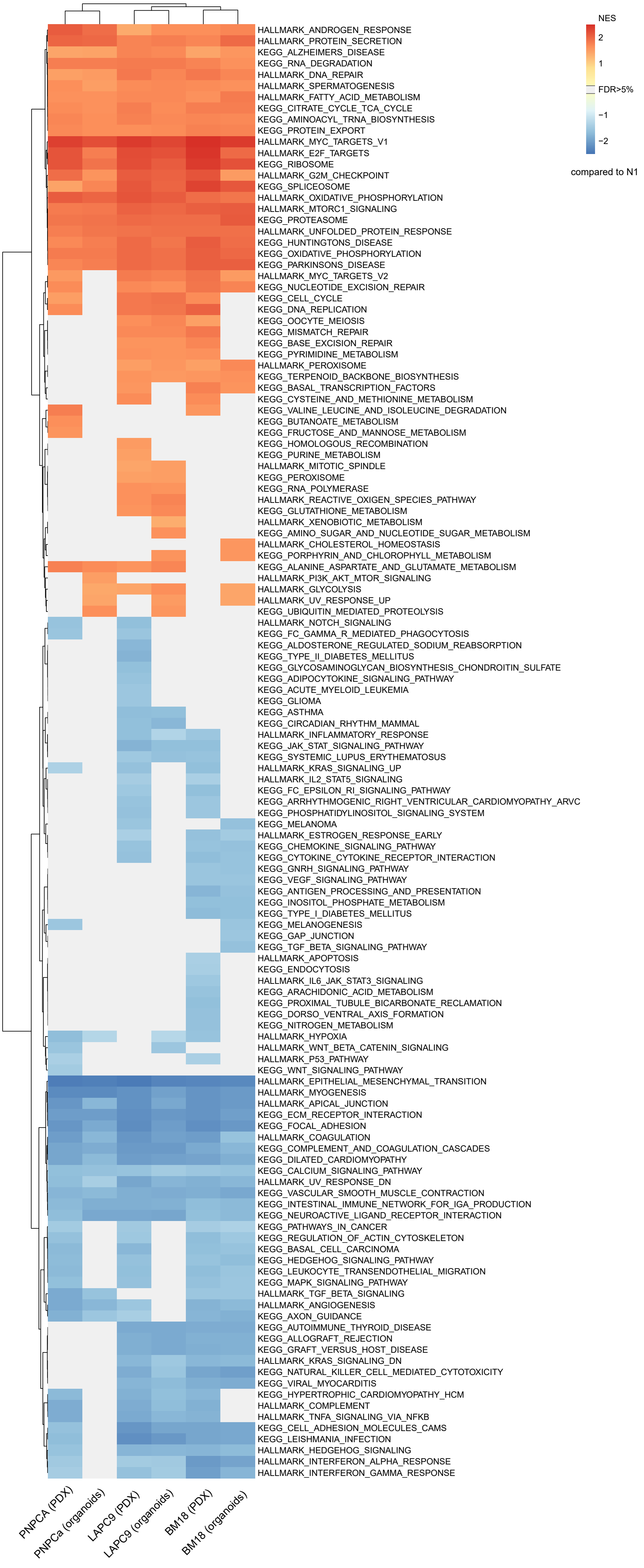

Sup.Figure 6. Gene set enrichment analysis (fGSEA) of PDXs and PDX-derived organoids, related to Fig.2

Enrichment scores of all Hallmark and KEGG (C2) pathways with FDR < 0.05 derived from differential expression analysis of each group of samples (PNPCa PDX N=3, BM18 PDX N=2, LAPC9 PDX N=2, PNPCa organoids N=2, BM18 organoids N=2, LAPC9 organoids N=2) vs. the non-carcinoma control tissue from PNPCa clinical sample (N1). Differential expression was inferred using the quasi-likelihood F-test based on a negative binomial dispersion model of raw counts and a design matrix (edgeR package). Gene set enrichment scores were inferred using the standard GSEA method (correlation-weighted Kolmogorov–Smirnov test, the normalized ES, and the false discovery rate calculation).

Supp. Fig. 7

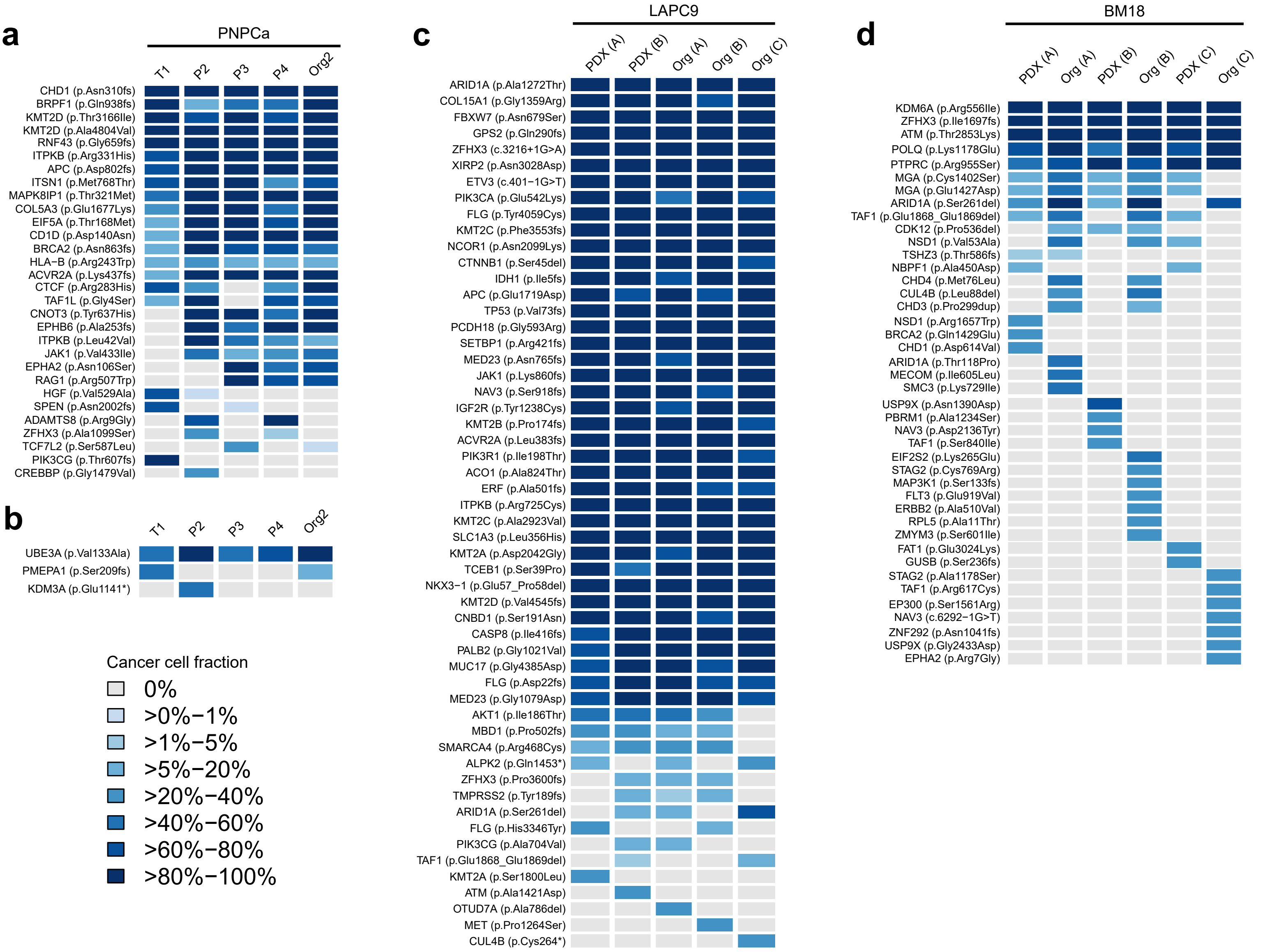

Supp.Figure 7. Somatic mutations in cancer genes in PCa PDX and PDX-derived organoids, related to Fig.2

a. Heatmap of cancer cell fraction of all identified non-synonymous somatic mutations in PNPCa in known cancer genes; amino acid position of the mutation is indicated next to the gene symbol in parentheses. **b.** Heatmap indicating cancer cell fraction of all non-synonymous somatic mutations in genes of the AR signaling pathway. **c.** Cancer cell fraction of non-synonymous somatic mutations in known cancer genes of LAPC9 PDX tissue and organoids and **d**. of BM18 PDX tissue and organoids. For **c-d**, mutations with a cancer cell fraction less than 20% that occurred in single samples were omitted.

a

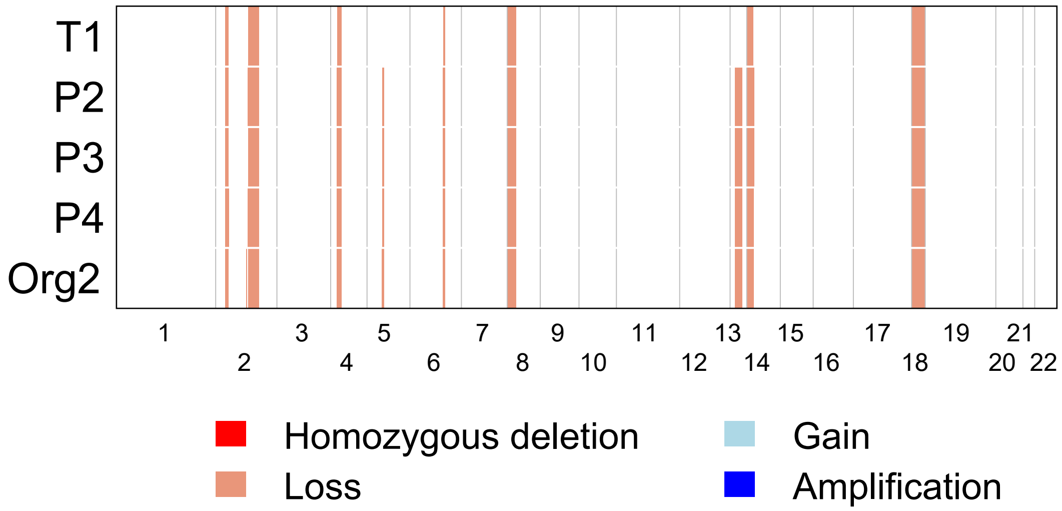

b

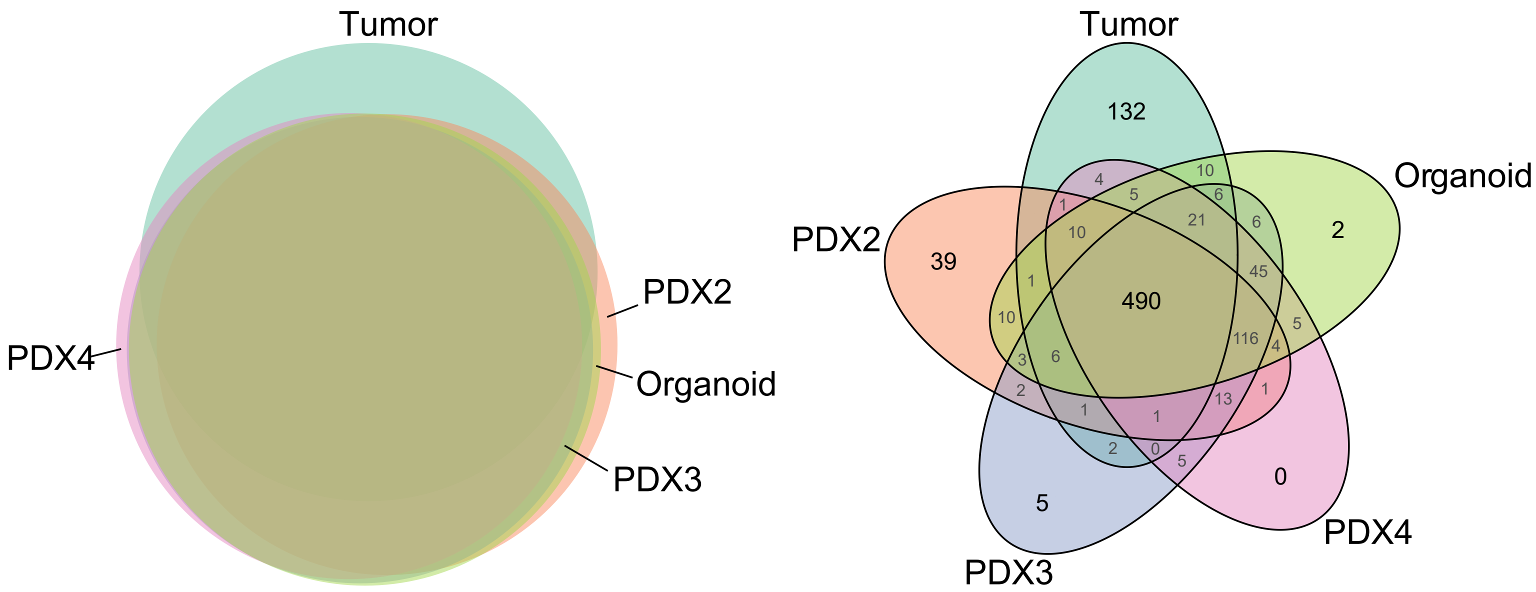

Sup.Figure 8. Genomic profile of PNPCa related to Fig.2

a. Genome-wide copy number profiles of genetic rearrangements and **b.** Euler (left) and Venn (right) diagram of the number of somatic mutations from whole exome sequencing of patient-derived material (primary tumor TUR-P ("T1")), PDX passages from PNPCa met (P2, P3, P4) and PDX (P4)-derived organoids (Org2).

Supp. Fig. 9

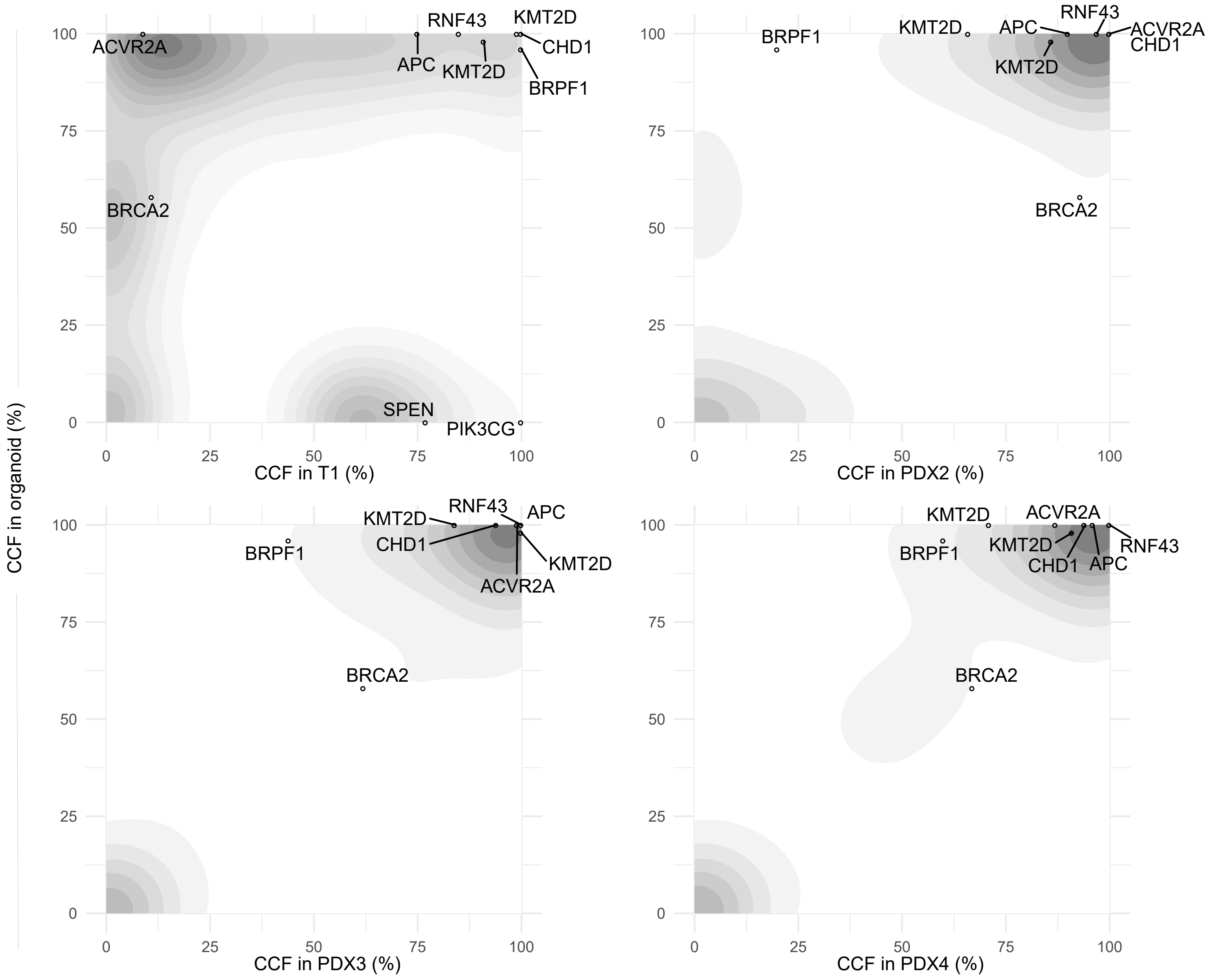

Supp.Figure 9. Density correlation plots of cancer cell fraction, related to Fig.2

Correlation plots of cancer cell fraction (CCF, %) in PNPCa model; organoid Org2 sample (y axis), versus P2 (PDX2), T1 (Tumor), P3 (PDX3) and P4 (PDX4).

Supp. Fig. 10

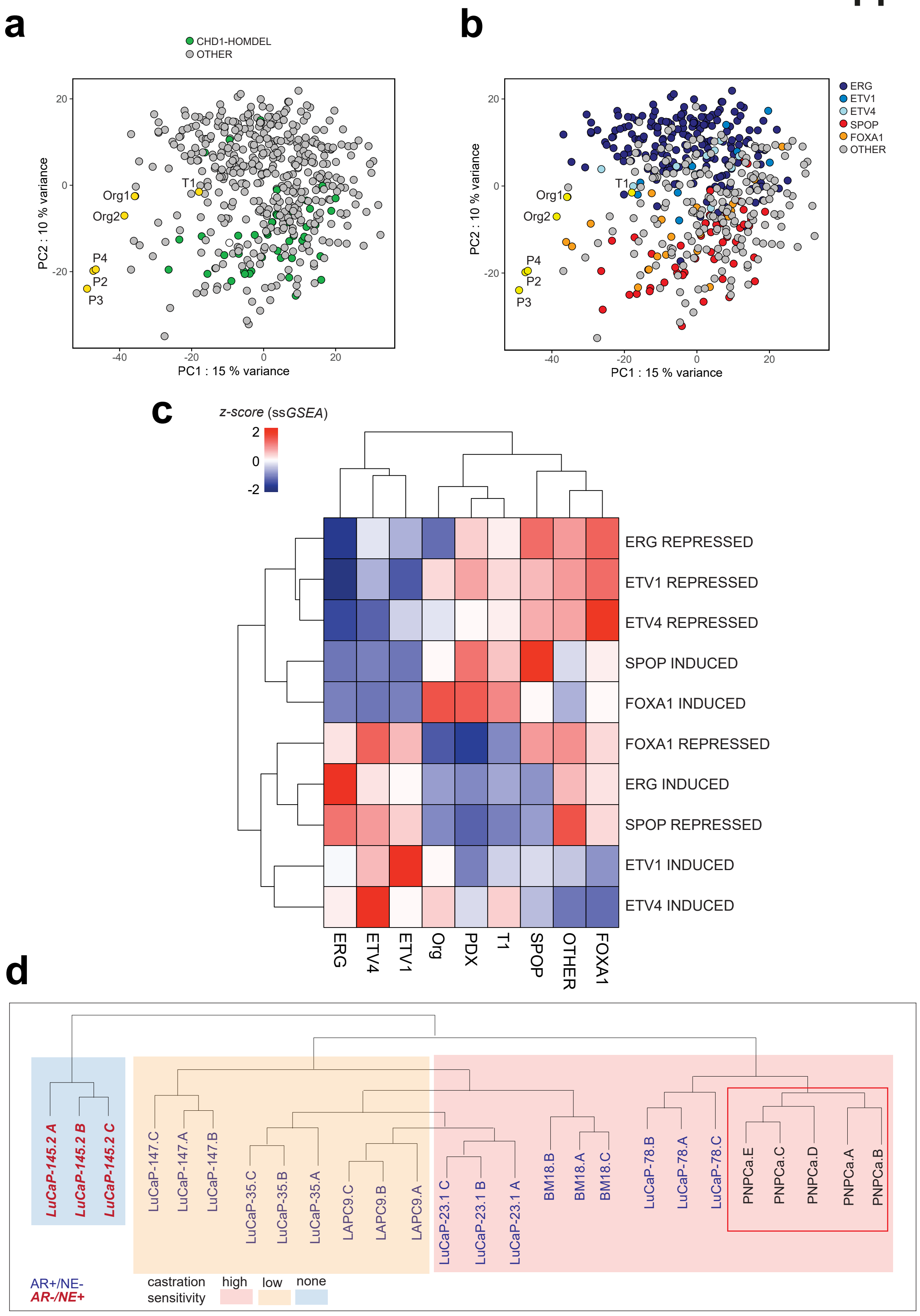

Supp.Figure 10. Transcriptomic landscape of PNPCa comparison to SPOP, FOXA1 and CHD1-like signatures, and hierarchical clustering to LuCaP subcohort, related to Fig.2

a. Principal component analysis (PCA) of TCGA gene expression data from 480 primary PCa tumors, classified based on CHD1 homozygous deletion and **b.** genetic subtype (SPOP, FOXA1, ETS rearrangements; ERG, ETV1, ETV4). **c.** Z score of single sample gene set enrichment analysis (ssGSEA) from the different genetic subgroups. N1, normal tissue; T1, primary tumor; P2-4, PNPCa PDX passage 2-4; Org1-2, PNPCa PDX-derived organoids passage 1-2. Others; cases with no mutations in SPOP, FOXA1, CHD1 and ETS groups. FOXA1 induced genes (n = 109), FOXA1 repressed genes (n = 183), ERG induced genes (n = 178), ERG repressed genes (n = 291), ETV1 induced genes (n = 9), ETV1 repressed genes (n = 25), ETV4 induced genes (n = 23), ETV1 repressed genes (n = 28). **d.** Hierarchical clustering of gene expression (RNA-seq) data from LuCaP PDX subcohort (LuCaP-78, -23.1, -35, -145, -147 with replicates indicated as A,B,C), PNPCa, BM18 and LAPC9. PNPCa A and B are replicates from higher passage (>10), PNPCa C, D and E are PDX passage p2, p3, p4 respectively as in Fig.2E. PNPCa samples are enclosed in a red box. AR; androgen receptor, NE; neuroendocrine status.

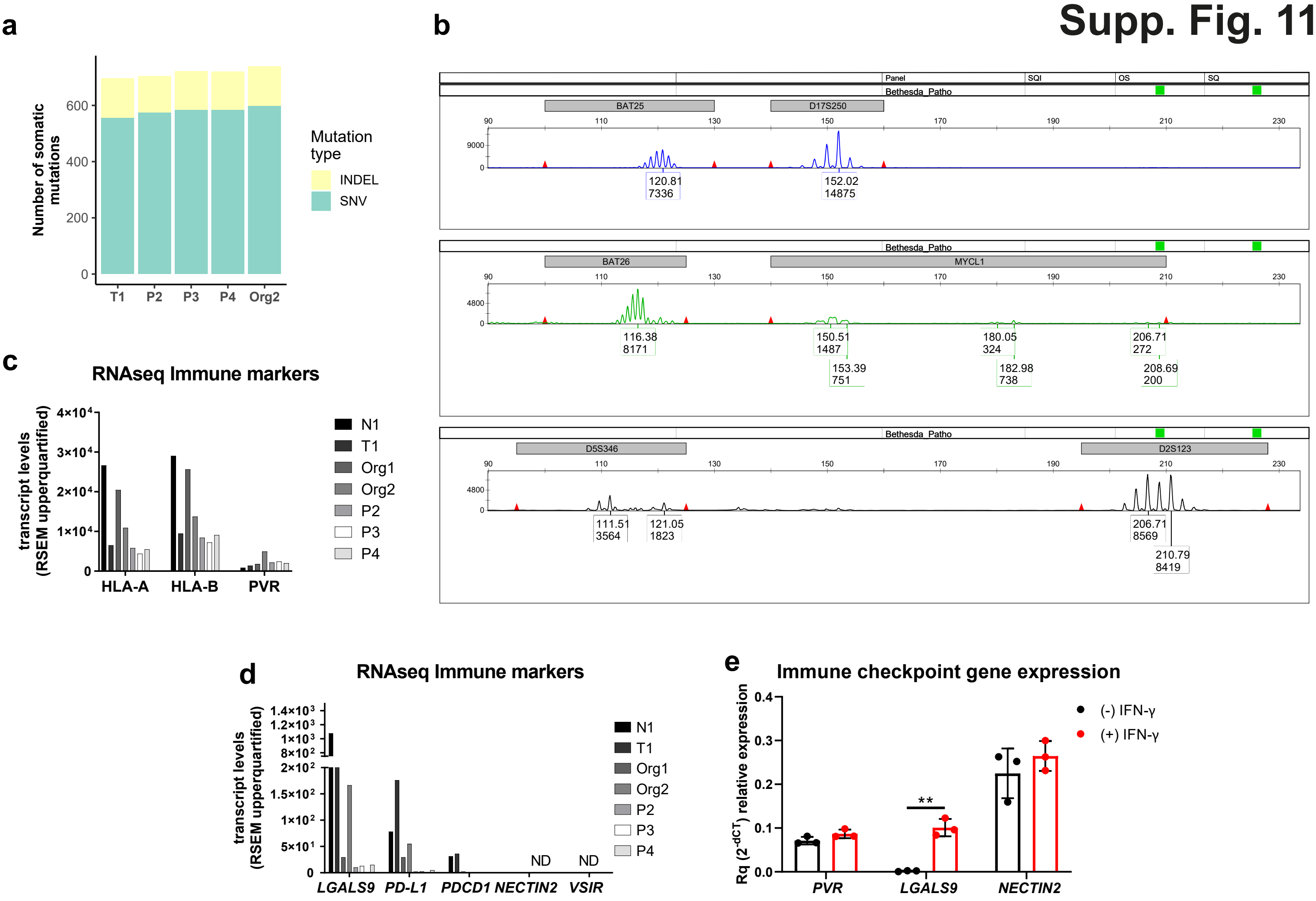

Supp.Figure 11. Confirmation of Microsatellite Instability of the primary tumor (T1), and immune marker expression, related to Fig.3

a. Number of somatic mutations divided into single nucleotide variants (SNVs) and insertions-deletions (*indels*) of the PNPCa models. **b.** MSI testing based on the Bethesda panel, which consists of six the loci BAT25, BAT26, MYCL1, D2S123, D5S346, and D17S250. Four out of the six loci contain repeats, classifying the tumor as MSI-high. Genomic DNA from the primary T1 tumor was obtained from high carcinoma- containing FFPE cores, as identified at pathological evaluation. **c,d.** Gene expression of markers related to immunosuppression (RSEM quantified transcript levels from RNA sequencing experiments). N=1 each. **e.** Gene expression levels of immune markers based on RT-qPCR results on PNPCa organoids RNA after 48 hours treatment with IFN-γ. Mean ± SD are reported, N=3 technical replicates, representative of two independent experiments. Two tailed t-test with Holm-Sidak method with alpha=0.05. **, adjusted p=0.0029.

Supp. Fig. 12

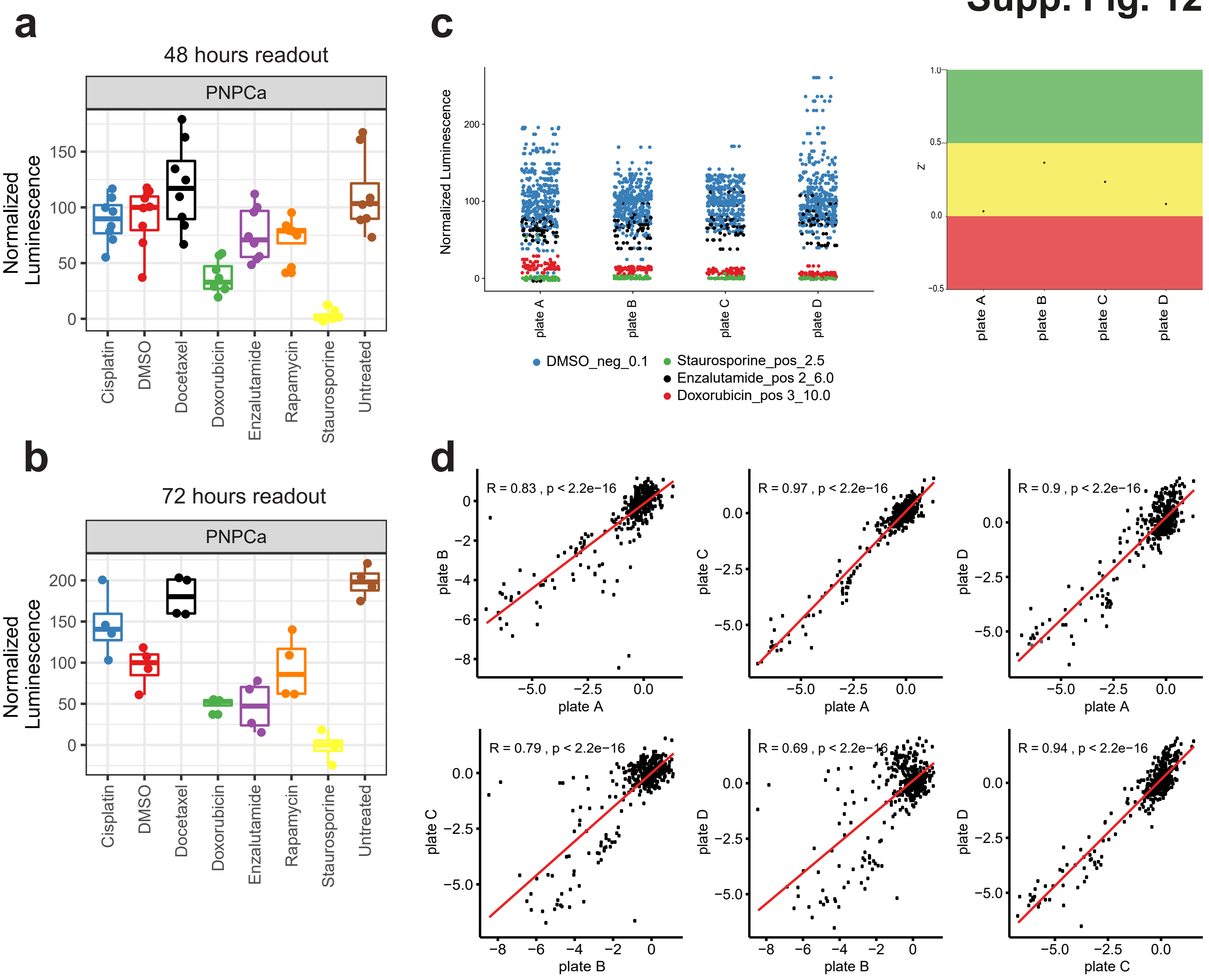

Supp.Figure 12. Optimization of Nexus pipeline for PNPCa PDX-derived organoids related to Fig. 4

a-b. Prescreen in response to standard-of-care compounds for assessment of positive controls and timing of drug exposure; 48 hours **(a)** and 72 hours **(b)**. Center line, box bounds and whiskers of box plots represent median, 1st/3rd quartile and 95% confidence interval respectively. N = 4 (a) or 8 (b) technical replicates from two independent experiments are shown. **c.** Viability assay assessing performance of different replicate plates (A-D) at 5000 cells/well for PNPCa organoids after 72 hours of drug treatment. Plots of all control conditions after NPI normalization per plate; positive controls (staurosporin) and negative controls (DMSO). Z prime factor > 0 indicates a marginal\good quality separation among positive and negative controls (right panel). **d.** Correlation plots of the $\log_2$ values between replicates obtained by luciferase measurements, proportional to cell viability, after 72h drug treatment.

Supp. Fig. 13

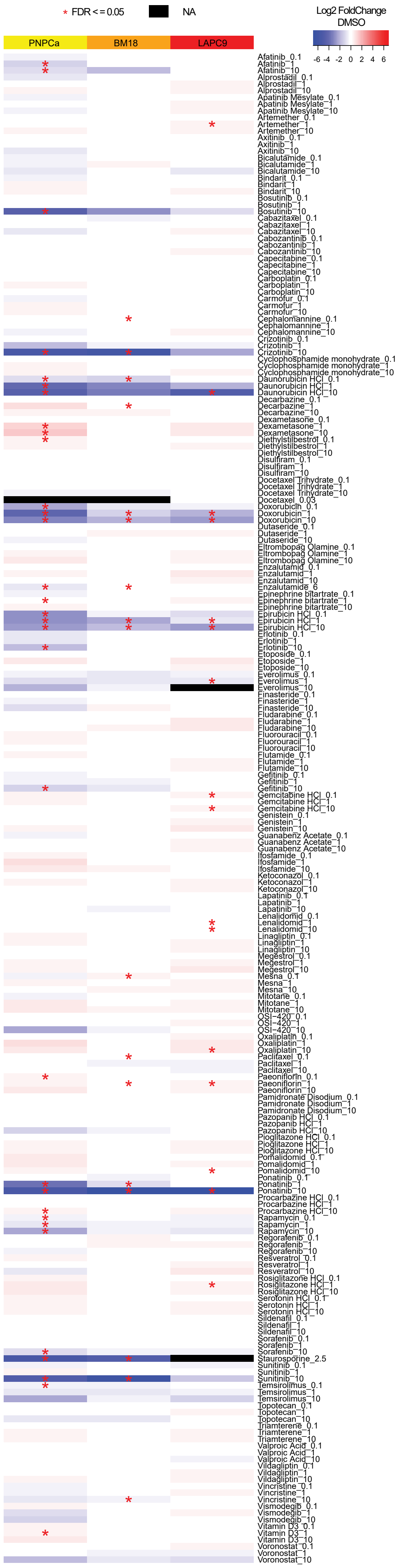

Supp.Figure 13. PNPCa, BM18 and LAPC9 organoid drug screens, related to Fig. 4

Heatmap of viability values of all tested compounds in the drug screen on PNPCa organoids (N=4 replicates, 72hrs) and BM18/LAPC9 organoids (N=3 each, 48hrs). Log2 fold change values relative to the DMSO control of each organoid model. Black segments, data not available.

Supp. Fig. 14

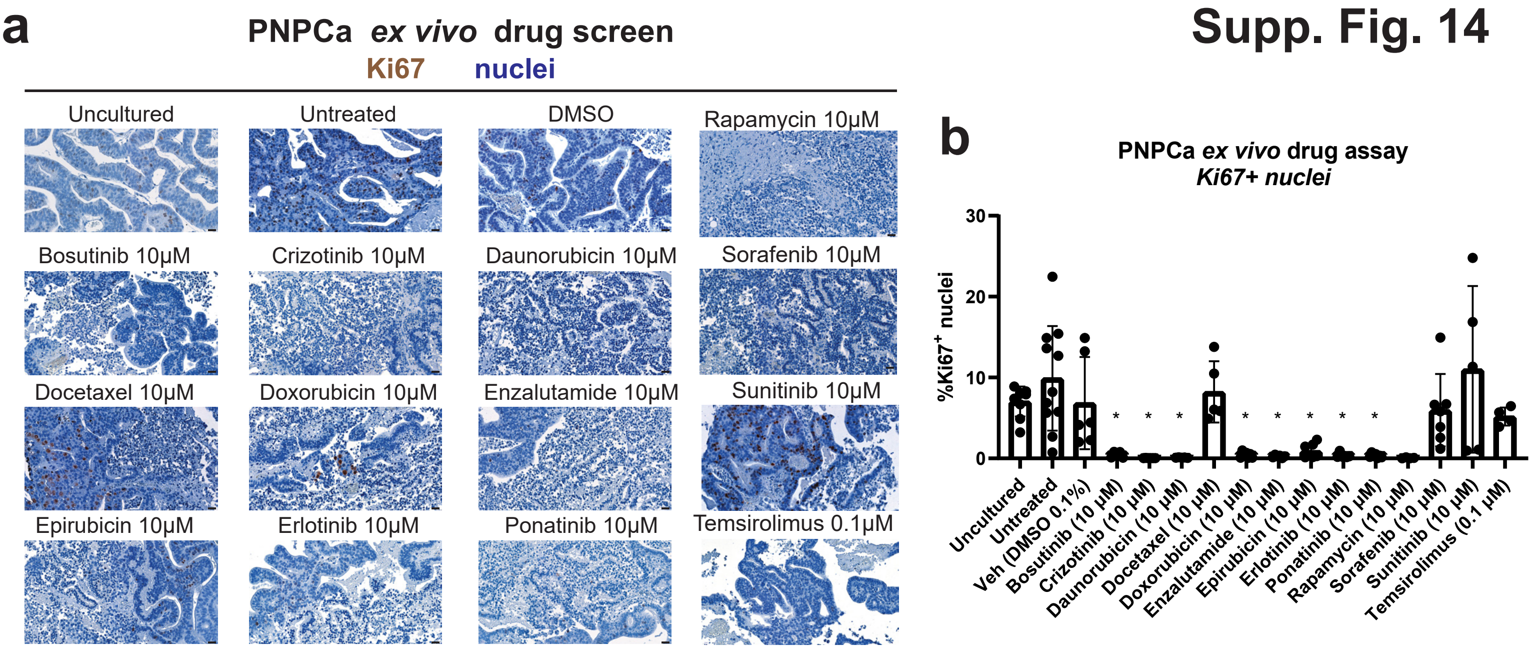

c

Compound	LAPC9			BM18			PNPCa		
	Dose	Ki67+ over total nuclei	p-value	Dose	Ki67+ over total nuclei	p-value	Dose	Ki67+ over total nuclei	p-value
Bosutinib	10 µM	0.3%	<0.0001				10 µM	0.4%	0.049
Crizotinib	10 µM	1.5%	0.0283				10 µM	0.0%	0.019
Daunorubicin	10 µM	2.4%	0.2335	1 µM	5.1%	0.0854	10 µM	0.1%	0.032
Docetaxel							10 µM	8.2%	0.999
Doxorubicin	1 µM	0.0%	<0.0001	10 µM	5.0%	0.0317	10 µM	0.4%	0.023
Enzalutamide				6 µM	1.4%	0.0007	10 µM	0.2%	0.026
Epirubicin	1 µM	0.0%	<0.0001				10 µM	0.9%	0.047
Erlotinib				10 µM	10.7%	0.107	10 µM	0.3%	0.029
Everolimus	1 µM	12.1%	0.0283						
Paclitaxel	1 µM	1.7%	0.2335						
Ponatinib	10 µM	0.5%	0.0002				10 µM	0.3%	0.021
Rapamycin	10 µM	7.4%	0.1726	10 µM	10.5%	0.1825	10 µM	0.0%	0.052
Sorafenib							10 µM	4.8%	0.999
Sunitinib	10 µM	0.2%	<0.0001				10 µM	10.3%	0.440
Temsirolimus	10 µM	9.2%	0.0707				0.1 µM	5.1%	0.999

Supp.Figure 14. Drug compound validation in *ex vivo* tissue slice assay for proliferation effects, related to Fig.4

a. Expression of Ki67 assessed by immunohistochemistry on PNPCa PDX tissue cultured *ex vivo* with selected drug compounds, used in the organoid screen. Hematoxylin counterstains the nuclei. Scale bars 50µm. **b.** Quantification of Ki67-positive cells, the count of positive nuclei was normalised over the total nuclei per field of view (%Ki67+ nuclei). Data are reported as mean ± SD of N≥4 distinct fields of view per condition across one representative experiment using 4 independent tumor samples. Scale bars 50µm. *, $p < 0.05$. **c.** Table of *ex vivo* tested compounds for each PDX model. One-way ANOVA with Dunnett correction.

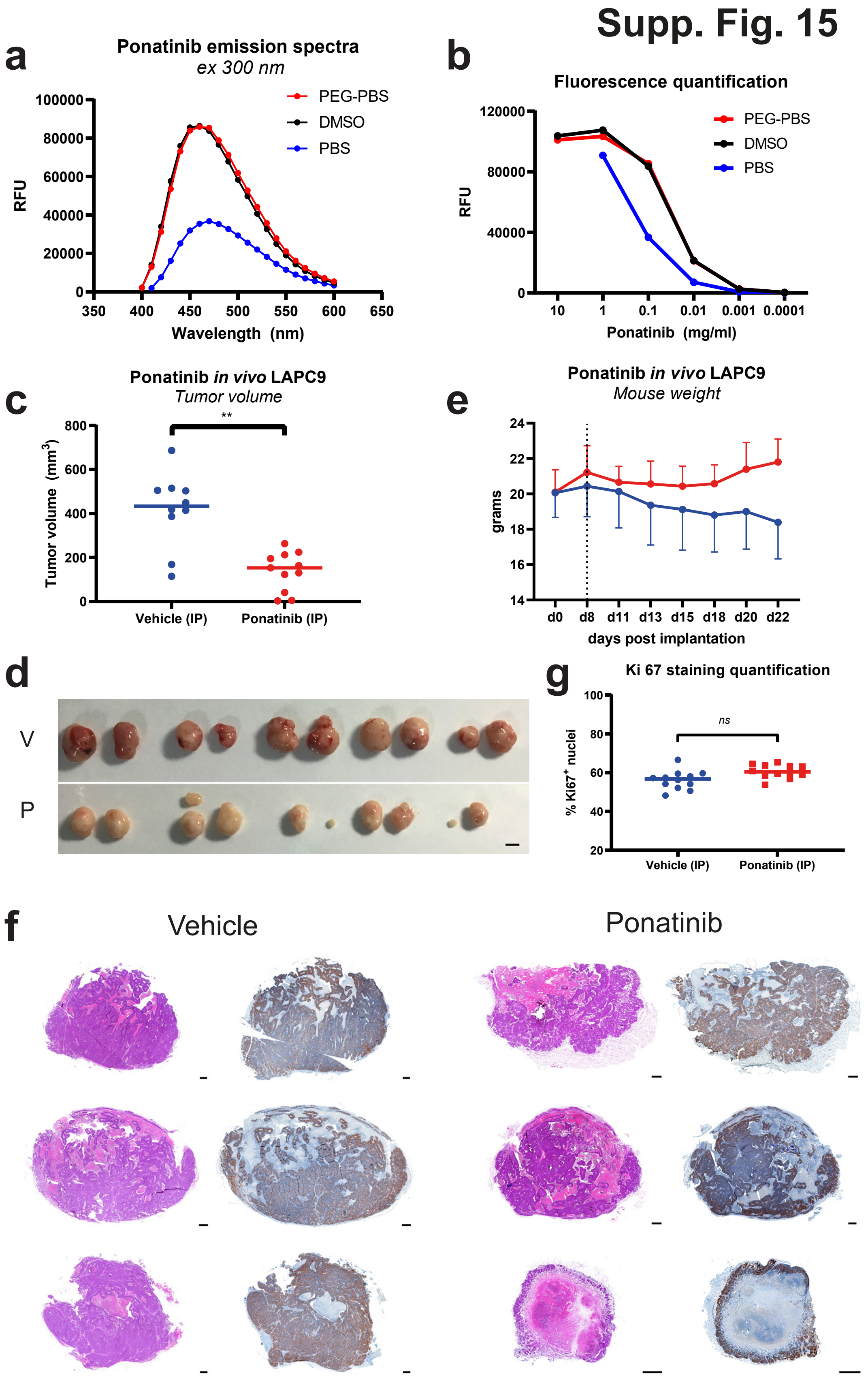

Supp.Figure 15. Ponatinib solubility and effects on LAPC9 *in vivo* growth, related to Fig. 4

a. Fluorescent emission spectra (ex 300 nm) of ponatinib dissolved at 0.1 mg/ml in PBS (blue), DMSO (black) or in a composite vehicle (PBS, 30%; PEG-300, 60%, DMSO, 10%). **b.** Fluorescent quantification (ex 300 nm) of ponatinib at different concentrations in the same vehicles as in A. For both A and B, the mean of 2 technical replicates is reported. Volume (**c**) and macroscopic image (**d**) of LAPC9 tumors at endpoint. Mean ± SD is reported, N=10 tumors examined over 5 engrafted animals, per group. Scale bars, 5 mm; Two-tailed nested t-test: **, p = 0.0026. **e.** Mouse weight during experiment, dotted line represent start of treatment with either ponatinib or vehicle. Mean ± SD is reported, N=5 engrafted mice per group. **f.** Additional hematoxylin and eosin staining (left) and Ki67 staining (right) of FFPE sections of LAPC9 tumors from mice treated with ponatinib or vehicle and not shown in Fig. 4. Scale bars, 0.5 mm. **g.** Quantification of Ki67 staining shown in panel F and in Fig. 4E. Two-tailed nested t-test was used to determine statistical significance. The average of 5 fields of view over N = 10 tumors across 5 mice per group is reported.

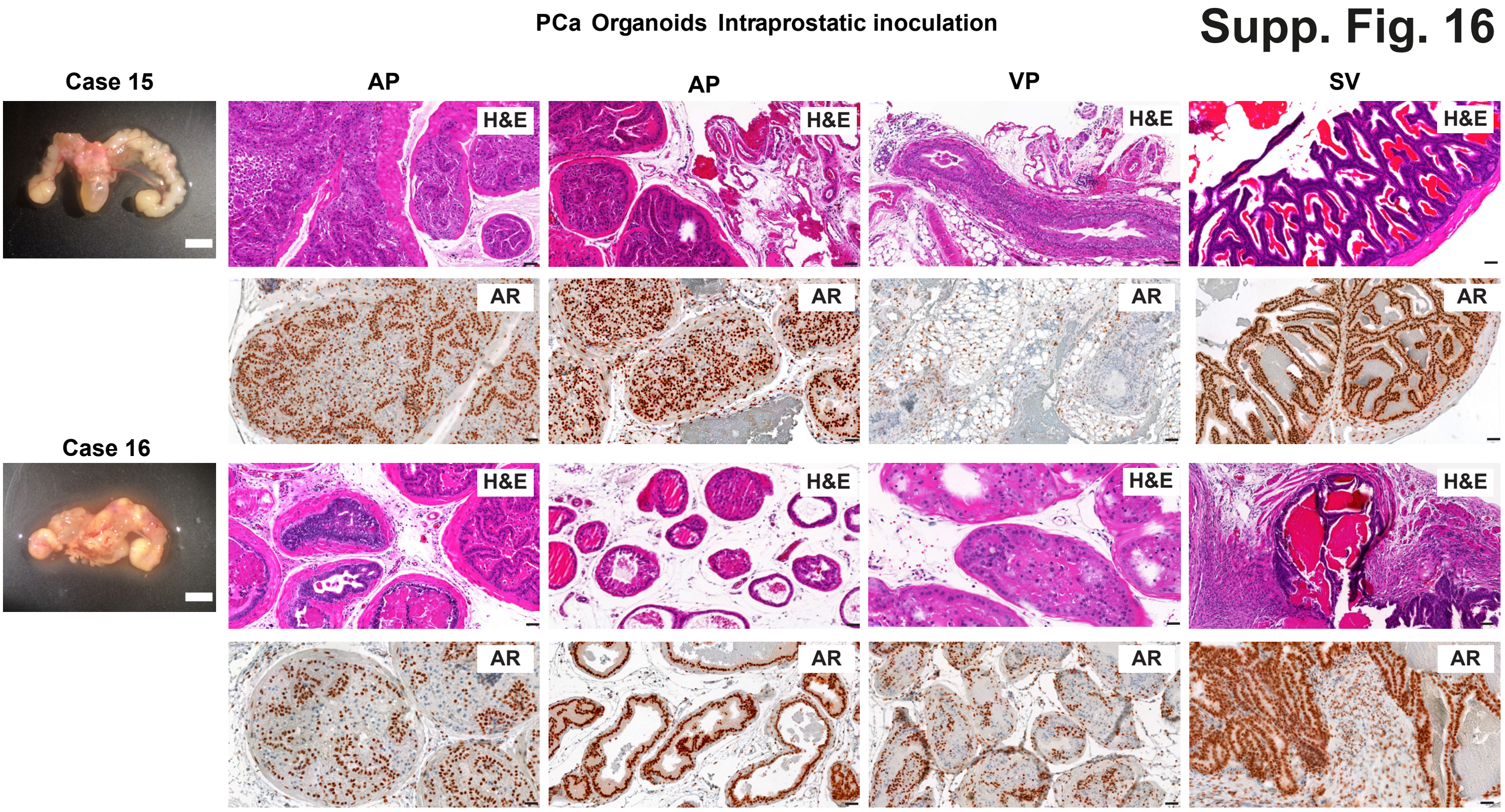

Supp.Figure 16. *In vivo* tumorigenicity of patient derived organoids, related to Fig. 5

Representative images of H&E and AR staining of prostatic tissues from intraprostatic inoculation of primary PCa organoids. Two representative cases are reported out of three engraftment experiments. AP; anterior prostate, VP; ventral prostate, SV, seminal vesicles.

Supp. Fig. 17

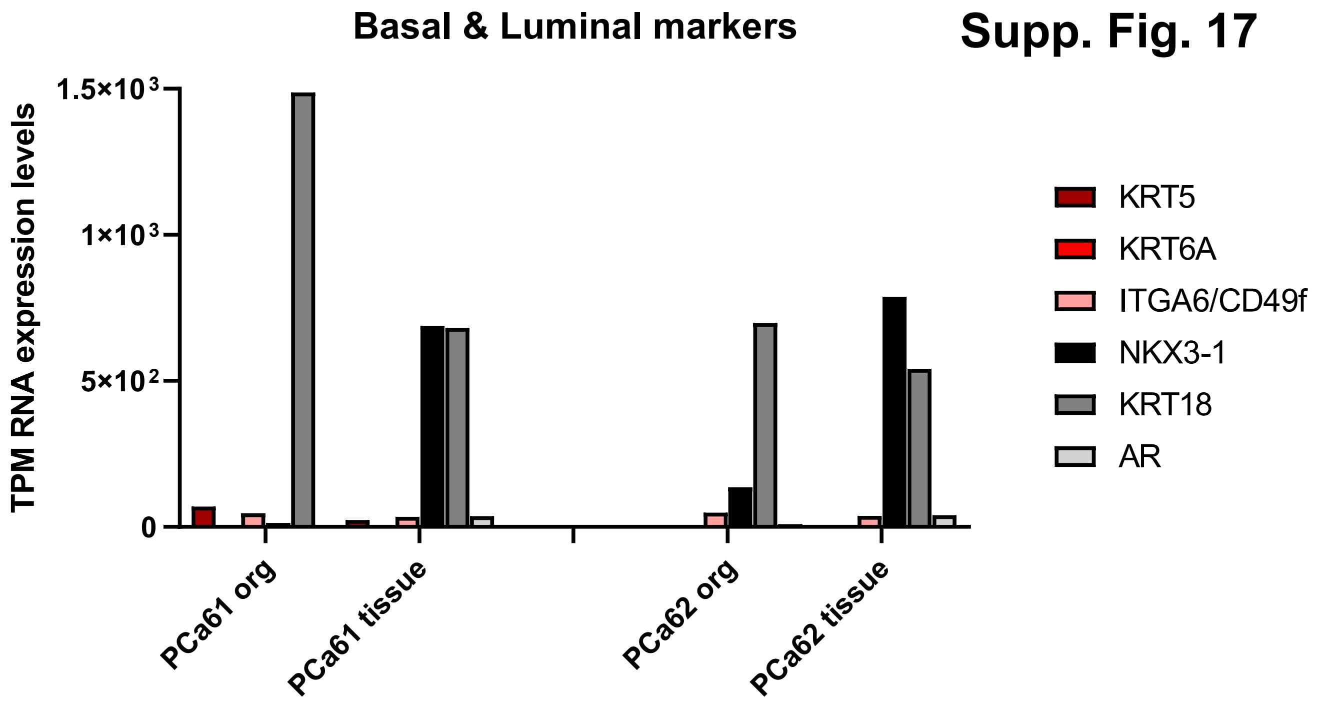

Supp.Figure 17. Expression of basal and luminal markers in patient-derived tumor tissues and organoids, related to Fig. 2

Gene expression (RNASeq, TPM counts) levels for within sample comparison of ratio of basal (*ITGA6*/CD49f, *KRT5*, *KRT6*) and luminal (*NKX3.1*, *AR*, *KRT18*) markers in different tumor tissues and corresponding organoids. N=1 each.

Supp. Fig. 18

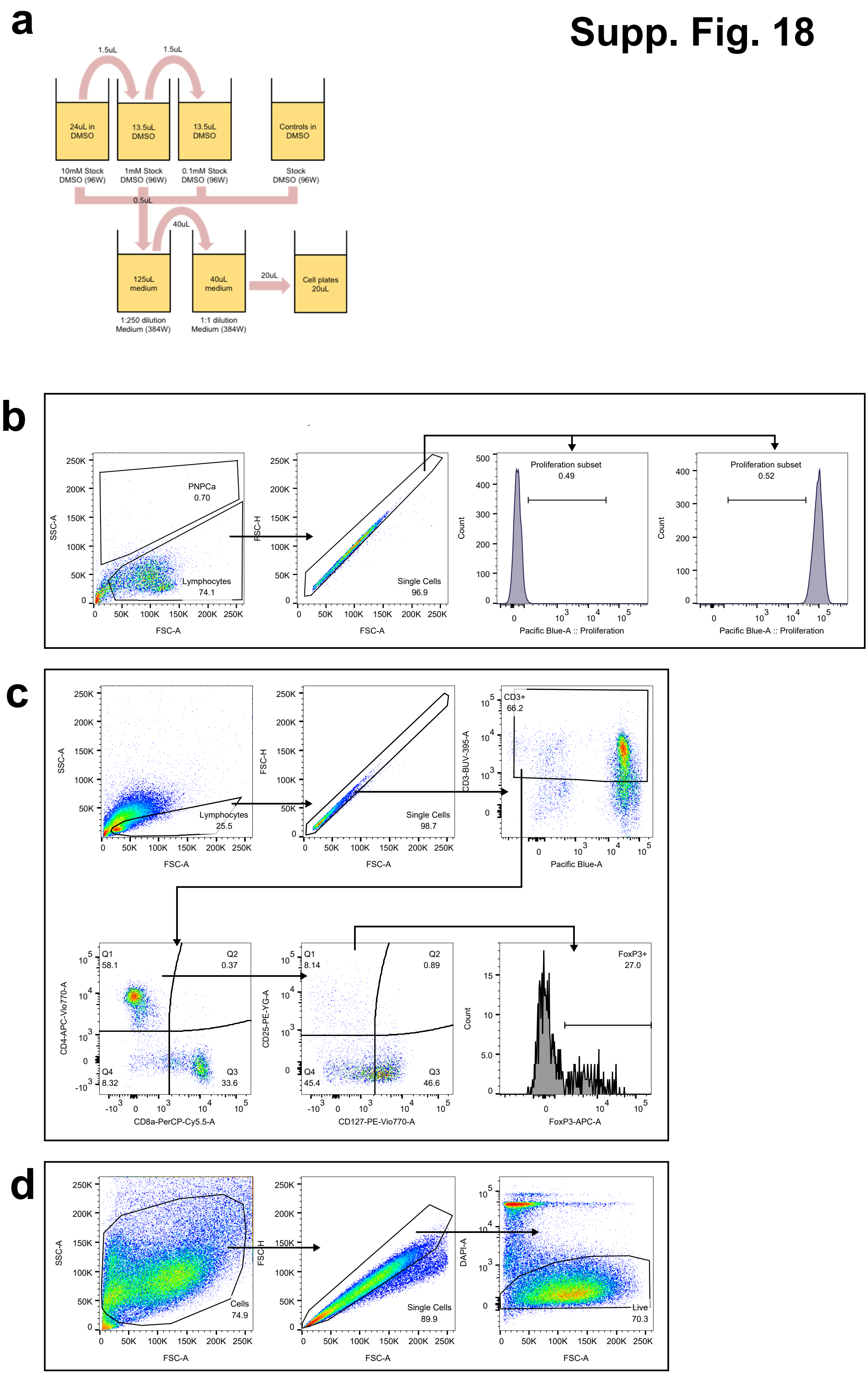

Supp.Figure 18. Automated drug dilution and addition to target plates, and gating strategy for flow cytometry experiments

a. Dilution steps of drug compounds from 96-well plates to 384-well plates performed at NEXUS Personalized Health Technologies. **b-d**. Gating strategies used for flow cytometry experiments. **b**. Gating strategy for lymphocytes proliferation presented in Fig. 3h of main text. Proliferation gate range was set using unstained CD3+ cells (Fig. 3b) and stained, unstimulated CD3+ cells (Fig. 4b). **c**. Gating strategy for regulatory T cells assay presented in Fig. 3i-j of main text. physical (SSC-A over FSC-A) and singlets gates (FSC-H over FSC-A) were followed by a CD3-positive gate and were used as parent for calculating the fraction of regulatory and of PD-1 positive T cells. CD4+CD25brCD127-FoxP3+ cells were selected by hierarchical gating for CD3, CD4 over CD8a, CD25 over CD127 dot plots and FoxP3 histogram plot. For PNPCa cells staining (panel D, referring to Fig. 1c in the main text), physical (SSC-A over FSC-A), singlets (FSC-H over FSC-A) and live cells gate (DAPI-A over FSC-A) were used as parent gates.

Supplementary Information (Tables)

Patient-derived xenografts and organoids model therapy response in prostate cancer

Sofia Karkampouna* and Federico La Manna*, et al.

Supplementary Table 1

Number of families	1
Number of comparisons per	43
Alpha	0.05

Compare each cell mean with the other cell mean in that row

Sidak's multiple comparison	Predicted (LS) mean	95.00% CI of diff.	Significant?	Summary	Adjusted P Value
Castrated - Castrated - Testost.					
0	4.00E-15	-2.699 to 2.699	No	ns	>0.9999
7	1.33E-15	-2.699 to 2.699	No	ns	>0.9999
14	8.88E-16	-2.699 to 2.699	No	ns	>0.9999
21	1.78E-15	-2.699 to 2.699	No	ns	>0.9999
28	-8.88E-16	-2.699 to 2.699	No	ns	>0.9999
35	-0.225	-2.924 to 2.474	No	ns	>0.9999
42	-0.5	-3.199 to 2.199	No	ns	>0.9999
48	-0.875	-3.574 to 1.824	No	ns	>0.9999
55	-0.05	-2.749 to 2.649	No	ns	>0.9999
62	-1.325	-4.024 to 1.374	No	ns	0.993
67	-1.325	-4.024 to 1.374	No	ns	0.993
74	-1.25	-3.949 to 1.449	No	ns	0.9976
81	-0.575	-3.274 to 2.124	No	ns	>0.9999
91	-0.575	-3.274 to 2.124	No	ns	>0.9999
102	0.625	-2.074 to 3.324	No	ns	>0.9999
108	-0.175	-2.874 to 2.524	No	ns	>0.9999
116	0.1667	-2.532 to 2.865	No	ns	>0.9999
123	-0.625	-3.324 to 2.074	No	ns	>0.9999
130	-0.5833	-3.282 to 2.115	No	ns	>0.9999
137	-0.5	-3.199 to 2.199	No	ns	>0.9999
144	-0.4167	-3.115 to 2.282	No	ns	>0.9999
151	-0.125	-2.824 to 2.574	No	ns	>0.9999
158	-1.78E-15	-2.699 to 2.699	No	ns	>0.9999
161	1.33E-15	-2.699 to 2.699	No	ns	>0.9999
165	0.3333	-2.365 to 3.032	No	ns	>0.9999
168	1.78E-15	-2.699 to 2.699	No	ns	>0.9999
172	-0.25	-2.949 to 2.449	No	ns	>0.9999
175	-1.33E-15	-2.699 to 2.699	No	ns	>0.9999
179	-0.2083	-2.907 to 2.490	No	ns	>0.9999
186	-0.625	-3.324 to 2.074	No	ns	>0.9999
189	-0.625	-3.324 to 2.074	No	ns	>0.9999
196	-0.8333	-3.532 to 1.865	No	ns	>0.9999
203	-1.333	-4.271 to 1.605	No	ns	0.9984
210	-2.5	-5.438 to 0.4379	No	ns	0.2179
217	-3.333	-6.271 to -0.3954	Yes	*	0.0105
224	-3.667	-6.605 to -0.7288	Yes	**	0.0025
231	-4	-6.938 to -1.062	Yes	***	0.0005
238	-4.5	-7.438 to -1.562	Yes	****	<0.0001
245	-5.333	-8.271 to -2.395	Yes	****	<0.0001
252	-5	-8.366 to -1.634	Yes	****	<0.0001
259	-6.5	-9.866 to -3.134	Yes	****	<0.0001
267	-8	-11.37 to -4.634	Yes	****	<0.0001
273	-9.5	-12.87 to -6.134	Yes	****	<0.0001

Test details	Predicted (LS) mean	Predicted (LS) mea	Predicted (LS) mean	SE of diff.	N1	N2	t	DF
Castrated - Castrated - Testost.								
0	8.88E-16	-3.11E-15	4.00E-15	0.8244	5	4	4.85E-15	286
7	0	-1.33E-15	1.33E-15	0.8244	5	4	1.62E-15	286
14	0	-8.88E-16	8.88E-16	0.8244	5	4	1.08E-15	286
21	0	-1.78E-15	1.78E-15	0.8244	5	4	2.16E-15	286
28	-8.88E-16	0	-8.88E-16	0.8244	5	4	1.08E-15	286
35	0.9	1.125	-0.225	0.8244	5	4	0.2729	286
42	1.5	2	-0.5	0.8244	5	4	0.6065	286
48	2.5	3.375	-0.875	0.8244	5	4	1.061	286
55	3.7	3.75	-0.05	0.8244	5	4	0.06065	286
62	4.3	5.625	-1.325	0.8244	5	4	1.607	286
67	6.8	8.125	-1.325	0.8244	5	4	1.607	286
74	6	7.25	-1.25	0.8244	5	4	1.516	286
81	4.8	5.375	-0.575	0.8244	5	4	0.6975	286
91	3.3	3.875	-0.575	0.8244	5	4	0.6975	286
102	2.5	1.875	0.625	0.8244	5	4	0.7582	286
108	1.7	1.875	-0.175	0.8244	5	4	0.2123	286
116	1.167	1	0.1667	0.8244	5	4	0.2022	286
123	0	0.625	-0.625	0.8244	5	4	0.7582	286
130	0.6667	1.25	-0.5833	0.8244	5	4	0.7076	286
137	0.5	1	-0.5	0.8244	5	4	0.6065	286
144	0.3333	0.75	-0.4167	0.8244	5	4	0.5054	286
151	1.33E-15	0.125	-0.125	0.8244	5	4	0.1516	286
158	-8.88E-16	8.88E-16	-1.78E-15	0.8244	5	4	2.16E-15	286
161	0	-1.33E-15	1.33E-15	0.8244	5	4	1.62E-15	286
165	0.3333	-8.88E-16	0.3333	0.8244	5	4	0.4044	286
168	0	-1.78E-15	1.78E-15	0.8244	5	4	2.16E-15	286
172	8.88E-16	0.25	-0.25	0.8244	5	4	0.3033	286
175	-1.33E-15	0	-1.33E-15	0.8244	5	4	1.62E-15	286
179	0.1667	0.375	-0.2083	0.8244	5	4	0.2527	286
186	-1.33E-15	0.625	-0.625	0.8244	5	4	0.7582	286
189	-2.22E-15	0.625	-0.625	0.8244	5	4	0.7582	286
196	0.5	1.333	-0.8333	0.8244	5	4	1.011	286
203	8.88E-16	1.333	-1.333	0.8974	5	3	1.486	286
210	0.3333	2.833	-2.5	0.8974	5	3	2.786	286
217	-1.11E-15	3.333	-3.333	0.8974	5	3	3.714	286
224	-8.88E-16	3.667	-3.667	0.8974	5	3	4.086	286
231	0.1667	4.167	-4	0.8974	5	3	4.457	286
238	0.1667	4.667	-4.5	0.8974	5	3	5.014	286
245	-1.78E-15	5.333	-5.333	0.8974	5	3	5.943	286
252	-8.88E-15	5	-5	1.028	5	2	4.863	286
259	-6.66E-15	6.5	-6.5	1.028	5	2	6.322	286
267	-7.11E-15	8	-8	1.028	5	2	7.781	286
273	0	9.5	-9.5	1.028	5	2	9.24	286

Supplementary Table 1. Statistical analysis *in vivo* tumor growth in subcutaneous PDX PNPCa model, related to Fig.1
Two-way ANOVA statistical test was performed on the tumor growth measurements among treatment groups; castrated versus castrated-testosterone in the different time points.

Supplementary Table 2

SAMPLE	MEAN TARGET COVERAGE
BM18-1-Organoids	88.323708
BM18-1-Tumor	117.680089
BM18-2-Organoids	109.371015
BM18-2-Tumor	117.048572
BM18-3-Organoids	107.088719
BM18-3-Tumor	116.873862
LAPC9-1-Organoids	114.966307
LAPC9-2-Organoids	76.999323
LAPC9-3-Organoids	116.123366
LAPC9-m5L (Tumor)	114.18574
LAPC9-m5R (Tumor)	108.821301
PNPCa N1	97.89533
PNPCa Org1	84.889893
PNPCa Org2	118.989748
PNPCa P2	109.014545
PNPCa P3	101.59251
PNPCa P4	99.498631
PNPCa T1	106.700317

Supplementary Table 2. Mean coverage WES
Mean target coverage per sample obtained by whole exome sequencing.

Supplementary Table 3

TUMOR_SAMPLE	NORMAL_SAMPLE	CHROM	POS	REF	ALT	GENE	HGVS_P	VAF
P080-Cx-T-RG	-	chr17	7578553	T	C	TP53	p.Tyr126Cys	0.48
P080-Cx-T-RG	-	chr22	30054193	G	C	NF2	p.Met205Ile	0.48
P080-Cx-T-RG	-	chr3	178952085	A	G	PIK3CA	p.His1047Arg	0.25
P080-Cx-T-TS	-	chr17	7578553	T	C	TP53	p.Tyr126Cys	0.16
P080-Cx-T-TS	-	chr22	30054193	G	C	NF2	p.Met205Ile	0.51
P080-Cx-T-TS	-	chr3	178952085	A	G	PIK3CA	p.His1047Arg	0.14
P133-CD-T-RG	-	chr19	10610235	C	CG	KEAP1	p.Ala159fs	1
P133-CD-T-TS	-	chr19	10610235	C	CG	KEAP1	p.Ala159fs	0.97
P133-CD-T-TS	-	chr19	42753345	G	A	ERF	p.Pro307Ser	0.11
P123P-C2-T-RG	P123-BL	chr10	123258075	C	T	FGFR2	p.Glu537Lys	0.0616246
P123P-C2-T-RG	P123-BL	chr14	38061184	TTGAAGCG(	C	FOXA1	p.Gln263_Cys268del	0.559211
P123P-C2-T-RG	P123-BL	chr5	67593331	G	T	PIK3R1	p.Glu701*	0.499459
P123P-C2-T-TS	P123-BL	chr14	38061184	TTGAAGCG(	C	FOXA1	p.Gln263_Cys268del	0.0468571
P123P-C2-T-TS	P123-BL	chr2	47643439	C	T	MSH2	p.Ser316Phe	0.0897436
P123P-C2-T-TS	P123-BL	chr5	67593331	G	T	PIK3R1	p.Glu701*	0.388024
P134-CB-T-RG	P134-BL	chrX	70349258	C	G	MED12	p.Leu1224Val	0.11
P134-CB-T-TS	P134-BL	chrX	70349258	C	G	MED12	p.Leu1224Val	0.6
P110-C1-T-RG	P110-BL	No Mutations	No Mutations	No Mutations	No Mutations	No Mutations	No Mutations	No Mutations
P110-C1-T-TS	P110-BL	chrX	70472574	C	A	ZMYM3	p.Glu178*	0.113924
P121-Cx-T-RG	P121-BL	No Mutations	No Mutations	No Mutations	No Mutations	No Mutations	No Mutations	No Mutations
P121-Cx-T-TS	P121-BL	chr1	11189845	G	T	MTOR	p.Phe1888Leu	0.0430248
P121-Cx-T-TS	P121-BL	chr17	37619268	G	T	CDK12	p.Arg315Ile	0.0862069
P121-Cx-T-TS	P121-BL	chr17	47696688	T	G	SPOP	p.Tyr87Ser	0.192382
P121-Cx-T-TS	P121-BL	chrX	123164923	T	TG	STAG2	p.Glu80fs	0.322702
Case61_C1	-	chr5	226103	C	T	SDHA	p.Arg188Trp	0.2
Case61_C1	-	chr22	30069391	CGGA	C	NF2	p.Glu422del	0.31
Case61_C1	-	chrX	70470327	A	G	ZMYM3	p.Phe345Ser	0.71
Case61_C1_org	-	chrX	70470327	A	G	ZMYM3	p.Phe345Ser	0.6
Case62_LNL	-	chr13	48934204	T	G	RB1	p.Leu220Arg	0.35
Case62_LNL_org	-	chr13	48934204	T	G	RB1	p.Leu220Arg	0.14
P108-C1-T-RG	P108-BL	No Mutations	No Mutations	No Mutations	No Mutations	No Mutations	No Mutations	No Mutations
P108-C1-T-TS	P108-BL	No Mutations	No Mutations	No Mutations	No Mutations	No Mutations	No Mutations	No Mutations
P120-Cx-T-RG	P120-BL	No Mutations	No Mutations	No Mutations	No Mutations	No Mutations	No Mutations	No Mutations
P120-Cx-T-TS	P120-BL	No Mutations	No Mutations	No Mutations	No Mutations	No Mutations	No Mutations	No Mutations
P125-Cx-T-RG	P125-BL	No Mutations	No Mutations	No Mutations	No Mutations	No Mutations	No Mutations	No Mutations
P125-Cx-T-TS	P125-BL	No Mutations	No Mutations	No Mutations	No Mutations	No Mutations	No Mutations	No Mutations

Supplementary Table 3. Ion Torrent sequencing on PDOs

List of somatic mutations identified by ion torrent sequencing on PDOs in comparison with their matched tissue. Gene name, chromosome position, type of mutation and variant allele fraction are indicated. TS; tissue, RG; organoids, BL; matched blood germline, T; tumor as defined by pathologist`s evaluation, Cx, number of core related to the prostatectomy position.

Supplementary Table 4

Sample	Age	Ethnicity	Sample type	Tissue	GS (at diagnosis)	pT Stage	Hormone ablation/ADT	2nd line	3rd line	4th line
Case P61	74	Caucasian	Tissue	Prostate (primary)	4+4	pT3b	-	-	-	-
Case P62	58	Caucasian	Biopsy	Lymph node met	4+5	pT4	-	-	-	-
Case P80	62	Caucasian	Tissue	Prostate (recurrence)	4+5	pT3b	Bicalutamide, Goserelin	GnRH antagonist	-	-
Case P82	68	Caucasian	Biopsy	Liver met	4+5	pT3b	Leuprorelin, Bicalutamide	Abiraterone, Prednisone, Denosumab	-	-
Case P89	49	Caucasian	Biopsy	Liver met	5+5	ND	Leuprorelin	Abiraterone	Docetaxel, Enzalutamide	Cabazitaxel
Case P108	69	Caucasian	Tissue	Prostate (primary)	3+4	pT3a	-	-	-	-
Case P110	78	Caucasian	Tissue	Prostate (primary)	5+4	pT3a	-	-	-	-
Case P120	74	Caucasian	Tissue	Prostate (primary)	3+4	pT3a	-	-	-	-
Case P121	70	Caucasian	Biopsy	Prostate (primary)	4+3	pT3a	-	-	-	-
Case P123	70	Caucasian	Biopsy	Prostate (primary)	4+4	pT3a	-	-	-	-
Case P125	73	Caucasian	Biopsy	Prostate (primary)	4+4	pT3b	Dutasteride, tamsulosin	-	-	-
Case P133	51	Caucasian	Tissue	Prostate (primary)	3+3	pT2c	Dutasteride	-	-	-
Case P134	63	Caucasian	Tissue	Prostate (primary)	3+4	pT2c	-	-	-	-

Supplementary Table 4. Patient characteristics

Clinical information of patient cases related to PDO drug screens (Fig.5E). Information on tissue site (primary or metastases), Gleason score at diagnosis, Hormone ablation or androgen deprivation therapy (ADT) and other treatments prior to surgical procedure (tissue collection for organoid derivation), are indicated.

Supplementary Table 5

Primer	Forward	Reverse
CD-274/ PD-L1 (human)	TGG CAT TTG CTG AAC GCA TTT	TGC AGC CAG GTC TAA TTG TTT T
HLA-A (human)	AGA GAC AGC GTG GTG AGT CAT	AGA GAC AGC GTG GTG AGT CAT
HLA-B (human)	ACA GCT CCG ATG ACC ACA AC	CAG CCG TAC ATG CTC TGG A
HPRT (human)	AGACTTTGCTTTCCTTGGTCAGG	GTCTGGCTTATATCCAACACTTCG
LGALS9 (human)	GGA CGG ACT TCA GAT CAC TGT	CCA TCT TCA AAC CGA GGG TTG
NECTIN 2 (human)	CAC TTG CGA GTT TGC CAC C	GCC ACT GTC GTA GGG TCC T
PD-1 (human)	ACG AGG GAC AAT AGG AGC CA	GGC ATA CTC CGT CTG CTC AG
PVR (human)	GGA CGG CAA GAA TGT GAC CT	GGT CGT GCT CCA ATT ATA GCC T
VSIR (human)	ACT GTG TGG TGT ACC CAT CCT	ATC CCT TGA ATG TTG CTG TCC
β-actin (human)	GCACAGAGCCTCGCCTT	CCTTGCACATGCCGGAG

Supplementary Table 5. Primer sequences used in this study

Supplementary Methods

Patient-derived xenografts and organoids model therapy response in prostate cancer

Sofia Karkampouna* and Federico La Manna* *et al.*

Organoid Maintenance, Passaging, Freezing and Thawing

For first passage after isolation, organoids start forming within 2-3 days- up to 1 week and the media is changed after the first 2-4 days, depending on organoid density. Twice weekly organoids must be either refreshed/ split or media must be added. Eventually some cells, such as fibroblasts, will adhere to the plate bottom while the organoids remain in suspension and are collected by simply pipetting the supernatant into basis media. For passaging organoids when density is high or their size is >150µm, the organoid suspension is collected in 15-ml Falcon tube, washed in basis medium in 220g, 3 min. If many floating non-viable single cells are present in the culture: centrifuge at low speed (10rcf) for 5 min, remove supernatant and wash with 4 ml Basis medium and centrifuge to pellet cells. Organoids are then incubated with TryPLE at 37°C for minimum 5 min to obtain single cells. When absolute single cell suspension is required for seeding exact No of cells use 22- or 23-gauge needle attached to a 1 mL syringe. Divide cell suspension in 2 tubes (expansion vs freezing). Spin down at 220rcf, 3 min, RT. After counting cells are seeded in 1:4 or 1:8 (depending on density) in fresh organoid media containing 10µM Y-27632-HCl inhibitor in ULA plates. For cryopreservation, organoids are washed in basis media (220rcf, 3 min) and dissociated in TrypLE as mentioned before, resuspend in organoid freezing media (50% fetal calf serum, 40% Advanced F12/DMEM basis medium, 10%DMSO and aliquoted in cryovials (approximately 10E5 cells per vial).

Immunofluorescence & Immunohistochemistry

FFPE sections (4µm) were deparaffinised and used for heat mediated antigen retrieval (citrate buffer pH 6, Vector labs). Sections were blocked for 30min, RT in 1%BSA in PBS-0.1%Tween20 before incubation with primary antibodies at 4°C overnight. Sections were washed once in PBS-0.1%Tween20 for 5min, then twice in PBS for 5min before incubation at RT for 90min with secondary antibody, diluted in PBS. Sections were then washed once in PBS for 5min, then incubated in a 1 µg/ml solution of 4′,6-diamidino-2-phenylindole (DAPI, Thermofisher scientific, D1306) in PBS for 10min. Sections were then washed twice in PBS for 5min and mounted using Prolong Gold anti-fade mounting medium (Thermofisher Scientific). The following antibodies were used:

Dilution	Antibody	Company	Catalog No
1to500	**CK5/6**	Chemicon Milipore	MAB1620
1to500	**AR**	abcam	ab133273
1to200	**CK8**	Thermo Fisher	MA1-06318
1to250	**Nkx3.1**	AthenaES	314
1to1200	**p63**	Abcam	ab124762

1to500	**p63**	BD Pharmingen	559951
1to400	**Ki67**	Gene Tex	GTX16667
1to500	**CD44**	BD Pharmingen	550988
1to250	**panCK**	DAKO	M0821
1to400	**PD-L1**	Cell signaling, E1L3N	13684
1to1000	**PSA**	DAKO	A0562
1to250	**Anti-mouse, AlexaFluor 488**	Life Technologies	A21202
1to250	**Anti-rabbit, AlexaFluor 555**	Life Technologies	A31572

For Immunohistochemistry, antigen retrieval was performed for 10 min in citrate buffer, followed by blocking of endogenous peroxidases in H_2O_2-NaN_3 and with swine serum for 15min. Primary antibody PSA was diluted 1to3000 in 5% swine serum in DAKO antibody diluent. Staining of rabbit primary antibodies was developed using the anti-rabbit EnVision+System-HRP (DAKO, K4002) following manufacturer's instructions. Staining of mouse primary antibodies was developed using the anti-mouse EnVision+System-HRP (DAKO, K4004) following manufacturer's instructions. Ki67 staining was quantified with ImageJ v.1.52 using a semi-automated method previously published [1].

Whole mount immunofluorescence staining of organoids

Organoids in suspension were collected by pipetting P1000 into 15ml Falcon tube and washed with 250 µl of 1xPBS (50 rcf, 3min). Following short fixation in 2% paraformaldehyde PFA at RT for 20 min, cells were spun down in 15ml falcon tube 50rcf, 3min. Organoids were washed 3x with 1xPBS/Glycine solution for 10 minutes each, gently rocking (2 rpm), spin 50rcf for 3 min to remove the supernatant. Buffer preparation 10x PBS/Glycine: 38.0g NaCl, 9.38g Na_2HPO_4 (sodium phosphate dibasic anhydrous), 2.07g NaH_2PO_4 (sodium phosphate monobasic), 37.5g glycine in 500ml total volume, adjust pH to 7.4 and filter sterilize. Organoids are then washed 3x with 1xIF Wash solution for 10 minutes each, gently rocking. Buffer preparation 10xIF wash: 38.0g NaCl, 9.38g Na_2HPO_4 (sodium phosphate dibasic anhydrous), 2.07g NaH_2PO_4 (sodium phosphate monobasic), 2.5g NaN_3, 5.0g Bovine Serum A (Fraction V), 10ml Triton X-100, 2.5ml Tween-20 in 500ml total volume, adjust pH to 7.4 and filter sterilize. Blocking is done in 1xIF wash buffer + 10% swine serum for 1 hour at RT. Aspirate block and incubate in primary antibody diluted in blocking buffer (300µl) in 1.5ml Eppendorf tube for overnight, gently rocking. For the washes IF wash was added and organoids were transferred to 15ml falcon tube for the washes.

Three washes with 1xIF Wash solution for 10 minutes each, gently rocking at RT. Spin 50rcf, 3min. Organoids were incubated with secondary antibodies AlexaFluor donkey anti-rabbit or anti-mouse (Invitrogen 1:250 dilution) in block buffer (1xIF wash + 10% swine serum) for 2 hours, gently rocking at RT. Two washes with IF Wash solution for 20 minutes each, gently rocking at RT. Incubate with DAPI (1µg/ml) in 1xPBS for 10 minutes, gently rocking at RT. Wash and resuspend in ~100µl PBS/IF wash. For imaging, organoids are transferred in 8well chamber slides (Nunc Labtek II Chamber #1.5 Coverglass system 155409). Antibodies for whole mount staining were used at 1:100 dilution.

Viability assay Organoids-Cell Titer Glo 3D assay

Organoids are dissociated in TrypLE, and seeded as single cells in ULA 96well plates (5000 cells in 100µl media, minimum 4 replicates per condition). After 1-3 days depending on the reformation of organoids, measure the volume of media left (after 48hours, remaining volume is 75µl). Organoid media is prepared containing the drug compounds (2x of final concentration). Add 75 µl media plus drug treatments on organoids and incubated for 48hrs. Organoid suspension is transferred (100µl) into opaque 96-well plates for luciferase measurement (Thermo Fisher Nuna, 0.5ml capacity, 267350). Equal volume of Cell Titer 3D Glo (Promega) is added, using a plate reader, the plate is subjected for 5min orbital shaking RT and proceed with the guidelines of the assay. Luminescent signal is measured at Tecan plate Reader Infinite Pro 2000.

Animals maintenance and housing conditions

Animal protocols and experimental procedures were approved by the Cantonal Veterinary Ethical Committee, Switzerland (license BE 55/16). Animals were housed in individually ventilated cages, on Aspen bedding at max 5 animals per cage and had unrestricted access to food and fresh water. Maintenance diet and fresh water used for animal feeding were sterilized by irradiation and autoclaving respectively, before administration. Ambient temperature was 20±2 °C, kept at constant humidity of 50±10% and on a 12-h light dark cycle with automatic light control.

***In vivo* organoid tumorigenicity assay**

Orthotopic transplantations of organoids were performed in seven-week old nude BALB/CBYJNUDE male mice. Organoids were dissociated using accutase™ (StemCell Technologies, 07920), washed in basis media, spun down (330g, 4min) and resuspend in organoid media. Dissociated organoids were orthotopically injected in both anterior prostatic lobes (10 µl cell suspension volume per injection) using a syringe and a 28-gauge needle. For subrenal implantations, eight-week old nude BALB/CBYJNUDE mice or five-week old NOD-*scid* IL2Rgamma^{null} (NSG) immunodeficient male mice were used. Organoids grown in

suspension were collected, washed and resuspend in 100% Matrigel (Growth Factor Reduced, Corning 356231). Two Matrigel plugs of 10 µl each, were implanted into the subrenal capsule and tissues were collected after 11-13 weeks.

In vivo treatment with ponatinib

Ponatinib powder was resuspended in DMSO at 100 mg/ml by vortexing and incubation in a water bath at 37° C for 30 minutes. Stock ponatinib solution was diluted to 10 mg/ml in PEG-300 (Sigma), aliquoted to single-use dose and stored at -20 °C. The day of injection, a single-dose of ponatininb was thawed and brought to 5 mg/ml with a diluent solution (40% PEG-300, 60% PBS), vortexed for 30 seconds and used within 1h, discarding any leftovers.

LAPC9 PDX were subcutaneously transplanted in ten 5-week old CB17/SCID male mice, one piece per flank. After 8 days, mice were randomly assigned to vehicle or ponatinib groups stratifying by weight and started receiving 10 mg/Kg of ponatinib or an equivalent volume of vehicle (5% DMSO, 65% PEG-300, 30% PBS) daily intraperitoneally, alternating injection sides every other day to minimize discomfort and inflammation. Mice were weighted and palpated every other day to monitor tumor growth. After 14 days of treatment, tumors were collected and processed for downstream analyses. Tumor size at endpoint was measured by caliper at the three major axes (X,Y,Z) and tumor volumes were calculated according to this formula: Volume= 4/3*π*a *x* b *x* c, with a=X/2, b=Y/2, c=Z/2.

Flow cytometry, Mixed Leukocyte Reaction (MLR) and regulatory T cells (T-reg) assay

Antibodies used for flow cytometry

Dilution	Antibody	Company	Catalog No	Label
1:50	CD36	Miltenyi Biotec	130-110-879	PE-Violet 770
1:25	CD146	BD Biosciences	563619	AlexaFluor-647
1:10	PSMA	Miltenyi Biotec	130-106-608	PE
1:100	E-Cadherin (CD324)	BD Biosciences	743715	BV 711
1:100	CD49f	BD Biosciences	740416	BV 605
1:100	CD44	BD Biosciences	744273	BUV 395
1:33	CD4	Miltenyi Biotec	130-109-533	APC-Violet 770
1:50	CD127	Miltenyi Biotec	130-113-977	PE-Violet 770
1:50	CD25	Miltenyi Biotec	130-115-628	PE
1:33	CD3	BD Biosciences	564000	BUV 395
1:20	PD-1	Biolegend	367412	FITC

1:200	CD8a	Biolegend	300924	PerCP-Cy 5.5
1:20	FoxP3	eBioscience	77-5776	APC

PE, R-phycoerythrin; BV, Brilliant Violet; BUV, Brilliant Ultraviolet; FITC, fluorescein isothiocyanate; APC, allophycocyanin.

Reagents, media, instruments and gating strategy

Buffy coat-derived cells were cultured in complete RPMI medium consisting of RPMI-1640 (Sigma-Aldrich, Germany) supplemented with 10% heat-inactivated FBS (Thermo Fisher Scientific, Switzerland), 1% Glutamax supplement and 1% penicillin/streptomycin (both by Thermo Fisher Scientific). Human Fc receptors were blocked using human FcR Blocking Reagent (Miltenyi Biotec) at 1:20 dilution in FACS wash for 10 minutes. Compensation controls were generated using AbC Total Antibody compensation beads kit (Thermofisher Scientific) following manufacturer's instructions. Compensation controls were acquired independently for every experiment.

DC differentiation medium was prepared by adding 100 ng/mL granulocyte-macrophage colony-stimulation factor (GM-CSF, Miltenyi Biotec) and 100 ng/mL IL-4 (Peprotech Ltd, UK) to complete RPMI medium. MLR medium was prepared by adding to complete RPMI medium 1% ITS supplement (Thermo Fisher Scientific), 100 µg/ml R-spondin 1, 50ng/mL EGF, 10 ng/mL FGF-10, 10 ng/mL Wnt-3a and 1ng/mL FGF-2. FACS wash was prepared with 0.5% low endotoxin BSA (Sera Laboratories International, UK), 2mM EDTA (Sigma-Aldrich) in PBS at pH 7.4. All data were acquired using an LSR-II instrument (BD Biosciences, USA) and a minimum of 50.000 events was acquired for each sample. Gating strategy for MLR assay, Treg assay and PNPCa cells staining is reported in **Supp.Fig.18B-D** respectively.

PNPCa cells staining

Digested PNPCa PDX cells were washed twice in FACS wash, then aliquoted in FACS tube at 1 milion/tube, FcR-blocked and directly stained at room temperature, protected from light, for 20 minutes with the following antibody mix: anti-CD44-APC, anti-E-Cadherin-BV711, anti-PSMA-PE, anti-CD49f-BV605, anti-CD36-PE-Violet-770, anti-CD146-BV711. The samples were then washed with FACS wash, resuspended in 0.5ml of FACS wash with 50ng/ml DAPI (Invitrogen) and incubated for 10 minutes at room temperature before acquisition.

Mononuclear cells isolation and DC generation

Buffy coats were obtained from healthy donors and were used to isolate mononuclear cells (MNC) by gradient centrifugation (Lymphoprep; 1.077 g/mL; Axis-Shield, UK). After separation, CD14+ monocytes and CD3+ cells were purified from total MNC by magnetic separation columns (Miltenyi Biotec, Germany), according to manufacturer's instructions. Monocyte-

derived DCs (moDCs) were generated by culturing CD14+ cells in DC differentiation medium for 5 days at 37°C in 5% CO_2. After differentiation, moDC were maturated by incubation for 2 days in DC differentiation medium supplemented with IL-6 (100 ng/mL, Peprotech), TNFα (25 ng/mL, Miltenyi Biotech), IL-1β (30 ng/mL, Miltenyi Biotech), and 1 μg/mL PGE2 (Tocris).

MLR and T-reg assay: CD3+ cells were labelled with CellTrace Violet (Thermo Fisher Scientific) according to manufacturer's instructions at a final concentration of 5 μM and plated at 100.000 cells/well in 96-well ultra-low attachment plates (Corning). As a positive control CD3+ cells were cocultured with mature DC (1:10 to CD3+ cells) and, where indicated, PNPCa organoids-derived cells were added at a ratio of 1:2 to CD3+ cells; all cells were cultured in 200 μl MLR medium per well. Plate was protected from light and incubated for 5 days at 37°C in 5% CO_2. As a negative control, stained and unstained CD3+ cells were cultured alone in MLR medium. For MLR assays, cells were collected at day 5, washed once in FACS wash and directly analysed by flow cytometry. For T-reg assay, cells were collected at day 5, washed in FACS wash FcR-blocked and then directly stained at room temperature, protected from light, for 20 minutes with the following antibody mix: anti-CD4-APC-Violet-770, anti-CD127-PE-Violet-770, anti-CD25-PE, anti-CD3-BUV-395, anti-PD-1-FITC, anti-CD8a-PerCP-Cy5.5. Cells were washed twice and then stained for FoxP3 using the FoxP3 Staining Buffer Set (eBioscience) and following manufacturer's instructions. A FoxP3-APC fluorescence minus one (FMO) control was prepared and analysed for each experiment for gate setting.

Whole exome sequencing

DNA extracted from FFPE (original patient material), frozen tissue (PDXs) or organoids (300ng) for the PNPCa model were sequenced using whole-exome sequencing. Whole-exome capture libraries were constructed after sample-shearing, end repair, and phosphorylation and ligation to barcoded sequencing adaptors. Ligated DNA was subjected to HaloPlex Exome (Agilent) as previously described [2]. Sequencing was performed using Illumina HiSeq 2500 (2 × 100 bp).

Sample preparation and hybridization capture for BM18, LAPC9 were based on the SureSelectXT Low Input Automated Target Enrichment for Illumina Paired-End Multiplexed Sequencing Version A0 (G9703-90010) using the Bravo Liquid Handling system. The Agilent SureSelectXT human all exon v7 capture library (5191-4006) was used. Given the lack of matched germline control, we sequenced additional whole blood DNA samples from eight individuals by the same workflow to serve as control. Clustering and DNA sequencing using the NovaSeq6000 was performed according to manufacturer's protocols. Mean amplicon coverages can be found in **SI Table 2.**

For the PNPCa model, sequence alignment was performed to the GRC37/hg19 reference using Burrows-Wheeler Aligner (BWA, v0.7.12) [3]. Somatic mutations and copy number alterations were determined according to the New York State Department of Health validated pipeline of EXaCT-1 clinical test [4], which accounts for the use of an amplicon-based WES assay, by comparison of each tumor sample with the matching control. The computational tool SPIA ensure that the samples from the same individual are analyzed [5].

For the BM18 and LAPC9 models, sequence reads were aligned to the reference human genome GRCh37 using BWA [3]. Local realignment, duplicate removal and base quality adjustment were performed using the Genome Analysis Toolkit (GATK, v3.6) [6] and Picard (http://broadinstitute.github.io/picard/). Somatic single nucleotide variants (SNVs) and small insertions and deletions (indels) were detected using MuTect (v1.1.4) [7] and Strelka (v1.0.15) [8], respectively, using the pool of normal as reference. We filtered out SNVs and indels outside of the target regions, those with variant allelic fraction (VAF) of <1% and/or those supported by <3 reads. We excluded variants for which the tumor VAF was <5 times that of the VAF of the pool of normal DNA sequenced and processed with the BM18 and LAPC9 samples, as well as all mutations found in the non-TCGA subset of the Exome Aggregation Consortium database v0.3.1 [9], and the Genome Aggregation Database (gnomAD v2.1.1, exome) [10]. We further excluded variants identified in at least two of a panel of 123 non-tumoral samples [11].

For PNPCa, BM18 and LAPC9, to remove false positive mutations that may derive from mouse gDNA, we extracted human-derived reads with the R package disambiguate [12]. Specifically, sequenced reads were aligned to the mouse reference genome GRCm38, which, together with the sequence reads aligned to the human reference genome, were used by disambiguate to extract human-derived sequence reads. We then used Genome Analysis Toolkit HaplotypeCaller [13] to interrogate the positions of all called mutations. Variants no longer detected among the human-derived sequence reads were removed.

To account for the presence of somatic mutations that may be present below the limit of sensitivity of somatic mutation callers, we used Genome Analysis Toolkit HaplotypeCaller[13] to interrogate the positions of all unique mutations in all tumor/organoids/PDX samples of a given tumor model to define the presence of additional mutations.

Allele-specific CNAs were identified using FACETS (v0.5.14) [14] as previously described [15], which performs a joint segmentation of the total and allelic copy ratio and infers allele-specific copy number states. Somatic mutations associated with the loss of the wild-type allele (i.e., loss of heterozygosity [LOH]) were identified as those were the lesser (minor) copy number state at the locus was 0. All mutations on chromosome X were considered to be associated with LOH. All gene amplifications and homozygous deletions were visually inspected using

plots of raw log2 and allelic copy ratios. Copy number states were collapsed to the gene level based on the median values to coding gene resolution based on all coding genes retrieved from the Ensembl (release GRCh37.p13).

The cancer cell fraction (CCF) of each mutation on the autosomes was inferred using the number of reads supporting the reference and the alternate alleles, and the segmented log2 ratio from WES as input for ABSOLUTE (v1.0.6) [16]. Solutions from ABSOLUTE were manually reviewed as recommended [16, 17]. Clonality analysis was done with Pyclone v.0.13.1 [18] . Clonal prevalence plot was done using the R package TimeScape v.1.12.0.

Cancer genes were annotated according to the cancer gene lists described by Kandoth et al. (127 significantly mutated genes) [19], Lawrence et al. (Cancer5000-S gene set) [20] and Armenia et al., (97 significantly mutated genes in PCa) [21]. Mutations affecting hotspot residues [22, 23] were annotated.

Decomposition of the mutational signature was performed using deconstructSigs [24] based on the set of 30 mutational signatures ("signature.cosmic," based on the signatures at https://cancer.sanger.ac.uk/cosmic/signatures [25, 26]. Signatures 6, 15, 20, 21 and 26 were considered to be associated with microsatellite instability. Microsatellite instability detection was performed using MSIsensor. Score ≥3.5 was indicated as MSI-H according to the original publication [27].

Targeted sequencing on the Ion torrent platform

Targeted sequencing of the PCa organoids (**Fig.5C;** PCa61, 62, 80, 133, 134, 123P, 110, 121, 108, 120, 125) and of the PNPCa PDX tumors (**Fig.1F**) was performed using a custom panel targeting the most frequently mutated genes in prostate cancers [28]. For organoids, sequence alignment was performed against the human reference genome hg19 using TMAP. For PDX tumors, sequence alignment was performed simultaneously against the human reference genome hg19 and the mouse reference genome GRCm38. For tumors and organoids with matched germline samples, somatic mutation calling was performed using PipeIT [29] which performs the initial variant calling step by Torrent Variant Caller (TVC, v5.0.3, Thermo Fisher Scientific) using low stringency variant calling parameters. PipeIT whitelists hotspot variants [23, 30] then filters out variants covered by fewer than 10 reads in either the tumor or the matched normal sample, supported by fewer than 8 reads or for which the tumor variant allele frequency (VAF) was <10 times that of the matched non-tumoral VAF. All variants were manually reviewed.

For tumors and organoids without matched germline samples, variant calling was performed by Torrent Variant Caller (TVC, v5.0.3, Thermo Fisher Scientific) using low stringency variant calling parameters. Mutations called were normalised using bcftools and left aligned using

GATK [13] . Hotspot variants [23, 30] were whitelisted. Variants found in homopolymer regions longer than 4 nucleotides, covered by fewer than 20 reads (total read depth at the locus), supported by fewer than 6 variant reads, with VAF < 0.1 were filtered out. Moreover, the variant allele must be observed in at least 3 forward reads and 3 reverse reads, with a strand bias < 0.2. Variants found in >0.5% of the population in any of the 1000 Genomes Project, the Exome Aggregation Consortium, the NHLBI Exome Sequencing Project and the Genome Aggregation Database, as well as variants with an allele fraction >90% or between 40% and 60% and found at any minor allele frequency in any of these four datasets were also filtered out. Variants found in a panel of 24 unmatched normal prostate tissues sequenced on the same platform were also removed. For samples derived from the PNPCa model, variants found in WES and covered in this targeted sequencing panel were genotyped using TVC to retrieve additional variants. All variants were manually reviewed.

RNA sequencing

RNA extracted from FFPE (original patient material PNPCa), frozen tissue (PDXs) or organoids from PNPCa, BM18, LAPC9 and PCa cases (300ng) were subjected to RNA sequencing. PNPCa specimens were prepared for RNA sequencing using TruSeq RNA Library Preparation Kit v2 or riboZero as previously described [31]. RNA integrity was verified using the Agilent Bioanalyzer 2100 (Agilent Technologies). cDNA was synthesized from total RNA using Superscript III (Invitrogen). Sequencing was then performed on GAII, HiSeq 2000, or HiSeq 2500.

For the BM18, LAPC9 and PCa RNASeq, the NEBNext Ultra II Directional RNA Library Prep Kit for Illumina was used to process the sample(s). The sample preparation was performed according to the protocol "NEBNext Ultra II Directional RNA Library Prep Kit for Illumina" (NEB #E7760S/L). Briefly, mRNA was isolated from total RNA using the oligo-dT magnetic beads. After fragmentation of the mRNA, a cDNA synthesis was performed. This was used for ligation with the sequencing adapters and PCR amplification of the resulting product. The quality and yield after sample preparation was measured with the Fragment Analyzer. The size of the resulting products was consistent with the expected size distribution (a broad peak between 300-500 bp). Clustering and DNA sequencing using the NovaSeq6000 was performed according to manufacturer's protocols. A concentration of 1.1 nM of DNA was used. Image analysis, base calling, and quality check was performed with the Illumina data analysis pipeline RTA3.4.4 and Bcl2fastq v2.20.

Sequence reads were aligned using STAR using the two-pass approach simultaneously to the human reference genome GRCh37 and the mouse reference genome GRCm38 [32].

Differential expression analysis was performed using the edgeR package [33]. Normalization was performed using the "TMM" (weighted trimmed mean) method and differential expression was assessed using the quasi-likelihood F-test, including a batch factor into the design matrix when required. Genes with FDR < 0.05 and > 2-fold were considered significantly differentially expressed. For gene set enrichment analysis (GSEA) from differential expression analysis, we ranked genes based on the F-statistics from differential expression analysis using the R package fgsea. Gene sets used were the C2 (KEGG gene sets), and H (hallmark gene sets) [34].

Fragment Analysis

MSI was assessed on the basis of the recommendations of the National Cancer Institute workshop on MSI, a panel of microsatellite loci (BAT25, BAT26, D2S123, D5S346, and D17S250) and two additional microsatellite markers (BAT40 and MYCL1) were used to determine MSI status [35]. PCR and fragment analysis were performed as previously described [36].

Transcriptomic signature profiling of SPOP, FOXA1, ETS genetic subgroups

For the analysis of transcriptomic signatures of the *SPOP*, *FOXA1* and ETS genetic subgroups [37], we retrieved gene-expression of 480 primary prostate cancer specimens from The Cancer Genome Atlas (TCGA). Raw-counts were retrieved using TCGABiolinks [38] package in R statistical environment. Normalization was performed using DESeq2 [39] pipeline, and data was ultimately transformed using variance stabilizing transformation. Genetic characterization of the tumors, in particular, mutations in *SPOP*, *FOXA1* and genetic rearrangements involving ETS transcription factors, was performed using annotations retrieved from cBioportal. We generated gene-signatures specific to each genetic alteration. To this purpose, we stratified the cohort in different subgroups (*FOXA1*, *SPOP*, *ERG*, *ETV1*, *ETV4*) and performed differential expression between these populations and all samples not affected by mutations in *SPOP/FOXA1* or ETS-rearrangements. We used a False Discovery Rate threshold of 0.001 and an absolute fold-change of 1. Gene-set activities were determined in individual patients using single sample gene-set enrichment analysis (ssGSEA) and subsequently averaged across groups.

Hierarchical clustering of LuCaP, BM18, LAPC9 and PNPCa PDXs

Unsupervised hierarchical clustering was performed in R statistical environment, using Euclidean distance measure and Ward linkage. Input matrix consisted in vst-normalized (DESeq2) [39] expression values. Analysis was performed on the basis of the 5000 most variable genes. Batch effect due to different sequencing centers has been corrected using the empirical Bayes regression implemented in ComBat, in the R package sva [40].

Software for Data analysis

Statistical analyses were performed using GraphPad Prism (v.8.3). Flow cytometry data were analysed with FlowJo (v.10.5). Immune fluorescence images were processed and exported using ZEN (blue edition, v.2.5). Histological stainings were scanned using 3D Histech CaseViewer (v.2.3).

Statistics and Reproducibility

For Fig. 1b, the reported micrographs are representative images from two independent staining experiments. For Fig. 1e, the reported micrographs are representative images of 3 independent tumors per group across two independent staining experiments. For Fig. 2a, micrographs are representative images from three organoid derivations per PDX (brightfield images, top panel), the reported immunofluorescence images are representative of two independent staining experiments. Micrograph presented in Fig. 3f are representative images of one experiment on one sample for each condition, evaluated using positive and negative technical controls. For Fig. 5a, the reported images are representative of three independent experiments. For Supplementary Fig. 1a-d, the reported micrographs are representative of nine independent experiments, summarized in panel 1e. Micrograph presented in Supplementary Fig. 2d-e are representative images from all the examined mice (3/3). Micrograph reported in Supplementary Fig. 4c-h and 4i-n are representative images of one experiment, performed on four and one animals respectively. For Supplementary Fig. 5b, micrographs in panels N1, T1 and Org2 are representative areas of one sample per condition; panels P2, P3 and P4 represent 3 independent biological replicates. For Supplementary Fig. 14a, micrographs are representative images of one out of three independent *ex vivo* experiments. For Supplementary Fig. 16, the micrographs reported are from two samples analysed out of three independent engraftment experiments.

References

1. La Manna, F., et al., *Dual-mTOR Inhibitor Rapalink-1 Reduces Prostate Cancer Patient-Derived Xenograft Growth and Alters Tumor Heterogeneity.* Frontiers in Oncology, 2020. **10**(1012).
2. Rennert, H., et al., *Development and validation of a whole-exome sequencing test for simultaneous detection of point mutations, indels and copy-number alterations for precision cancer care.* Npj Genomic Medicine, 2016. **1**: p. 16019.
3. Li, H. and R. Durbin, *Fast and accurate short read alignment with Burrows-Wheeler transform.* Bioinformatics, 2009. **25**(14): p. 1754-60.
4. Rennert, H., et al., *Development and validation of a whole-exome sequencing test for simultaneous detection of point mutations, indels and copy-number alterations for precision cancer care.* NPJ Genom Med, 2016. **1**.
5. Demichelis, F., et al., *SNP panel identification assay (SPIA): a genetic-based assay for the identification of cell lines.* Nucleic Acids Res, 2008. **36**(7): p. 2446-56.
6. McKenna, A., et al., *The Genome Analysis Toolkit: a MapReduce framework for analyzing next-generation DNA sequencing data.* Genome research, 2010. **20**(9): p. 1297-1303.
7. Cibulskis, K., et al., *Sensitive detection of somatic point mutations in impure and heterogeneous cancer samples.* Nature Biotechnology, 2013. **31**: p. 213.
8. Saunders, C.T., et al., *Strelka: accurate somatic small-variant calling from sequenced tumor–normal sample pairs.* Bioinformatics, 2012. **28**(14): p. 1811-1817.
9. Lek, M., et al., *Analysis of protein-coding genetic variation in 60,706 humans.* Nature, 2016. **536**: p. 285.
10. Karczewski, K.J., et al., *The mutational constraint spectrum quantified from variation in 141,456 humans.* Nature, 2020. **581**(7809): p. 434-443.
11. Nuciforo, S., et al., *Organoid Models of Human Liver Cancers Derived from Tumor Needle Biopsies.* Cell Reports, 2018. **24**(5): p. 1363-1376.
12. Ahdesmaki, M.J., et al., *Disambiguate: An open-source application for disambiguating two species in next generation sequencing data from grafted samples.* F1000Res, 2016. **5**: p. 2741.
13. McKenna, A., et al., *The Genome Analysis Toolkit: a MapReduce framework for analyzing next-generation DNA sequencing data.* Genome Res, 2010. **20**(9): p. 1297-303.
14. Shen, R. and V.E. Seshan, *FACETS: allele-specific copy number and clonal heterogeneity analysis tool for high-throughput DNA sequencing.* Nucleic acids research, 2016. **44**(16): p. e131-e131.
15. Piscuoglio, S., et al., *The Genomic Landscape of Male Breast Cancers.* Clinical cancer research : an official journal of the American Association for Cancer Research, 2016. **22**(16): p. 4045-4056.
16. Carter, S.L., et al., *Absolute quantification of somatic DNA alterations in human cancer.* Nature biotechnology, 2012. **30**(5): p. 413-421.
17. Landau, D.A., et al., *Evolution and impact of subclonal mutations in chronic lymphocytic leukemia.* Cell, 2013. **152**(4): p. 714-726.
18. Roth, A., et al., *PyClone: statistical inference of clonal population structure in cancer.* Nature methods, 2014. **11**(4): p. 396-398.
19. Kandoth, C., et al., *Mutational landscape and significance across 12 major cancer types.* Nature, 2013. **502**(7471): p. 333-339.
20. Lawrence, M.S., et al., *Discovery and saturation analysis of cancer genes across 21 tumour types.* Nature, 2014. **505**(7484): p. 495-501.
21. Armenia, J., et al., *The long tail of oncogenic drivers in prostate cancer.* Nat Genet, 2018. **50**(5): p. 645-651.

22. Chang, M.T., et al., *Identifying recurrent mutations in cancer reveals widespread lineage diversity and mutational specificity.* Nature biotechnology, 2016. **34**(2): p. 155-163.
23. Gao, J., et al., *3D clusters of somatic mutations in cancer reveal numerous rare mutations as functional targets.* Genome medicine, 2017. **9**(1): p. 4-4.
24. Rosenthal, R., et al., *DeconstructSigs: delineating mutational processes in single tumors distinguishes DNA repair deficiencies and patterns of carcinoma evolution.* Genome biology, 2016. **17**: p. 31-31.
25. Nik-Zainal, S., et al., *Landscape of somatic mutations in 560 breast cancer whole-genome sequences.* Nature, 2016. **534**(7605): p. 47-54.
26. Alexandrov, L.B., et al., *Signatures of mutational processes in human cancer.* Nature, 2013. **500**(7463): p. 415-421.
27. Niu, B., et al., *MSIsensor: microsatellite instability detection using paired tumor-normal sequence data.* Bioinformatics, 2014. **30**(7): p. 1015-6.
28. Salami, S.S., et al., *Transcriptomic heterogeneity in multifocal prostate cancer.* JCI Insight, 2018. **3**(21).
29. Garofoli, A., et al., *PipeIT: A Singularity Container for Molecular Diagnostic Somatic Variant Calling on the Ion Torrent Next-Generation Sequencing Platform.* The Journal of Molecular Diagnostics, 2019. **21**(5): p. 884-894.
30. Chang, M.T., et al., *Identifying recurrent mutations in cancer reveals widespread lineage diversity and mutational specificity.* Nature Biotechnology, 2015. **34**: p. 155.
31. Beltran, H., et al., *Whole-Exome Sequencing of Metastatic Cancer and Biomarkers of Treatment Response.* JAMA Oncol, 2015. **1**(4): p. 466-74.
32. Dobin, A., et al., *STAR: ultrafast universal RNA-seq aligner.* Bioinformatics (Oxford, England), 2013. **29**(1): p. 15-21.
33. Nikolayeva, O. and M.D. Robinson, *edgeR for differential RNA-seq and ChIP-seq analysis: an application to stem cell biology.* Methods Mol Biol, 2014. **1150**: p. 45-79.
34. Liberzon, A., et al., *Molecular signatures database (MSigDB) 3.0.* Bioinformatics, 2011. **27**(12): p. 1739-40.
35. Umar, A., et al., *Revised Bethesda Guidelines for hereditary nonpolyposis colorectal cancer (Lynch syndrome) and microsatellite instability.* J Natl Cancer Inst, 2004. **96**(4): p. 261-8.
36. Kishore, S., et al., *3'-UTR poly(T/U) tract deletions and altered expression of EWSR1 are a hallmark of mismatch repair-deficient cancers.* Cancer Res, 2014. **74**(1): p. 224-34.
37. Barbieri, C.E., et al., *Exome sequencing identifies recurrent SPOP, FOXA1 and MED12 mutations in prostate cancer.* Nat Genet, 2012. **44**(6): p. 685-9.
38. Colaprico, A., et al., *TCGAbiolinks: an R/Bioconductor package for integrative analysis of TCGA data.* Nucleic Acids Research, 2015. **44**(8): p. e71-e71.
39. Love, M.I., W. Huber, and S. Anders, *Moderated estimation of fold change and dispersion for RNA-seq data with DESeq2.* Genome Biology, 2014. **15**(12): p. 550.
40. Leek, J.T., et al., *The sva package for removing batch effects and other unwanted variation in high-throughput experiments.* Bioinformatics (Oxford, England), 2012. **28**(6): p. 882-883.
