# Supplementary material for: Patient-derived xenografts and organoids model therapy response in prostate cancer
Source: Nat Commun. 2021 Feb 18;12:1117. doi: 10.1038/s41467-021-21300-6 (PMC7892572; doi:10.1038/s41467-021-21300-6)
Supplement: Supplementary file 7 — Reporting Summary [file 41467_2021_21300_MOESM7_ESM.pdf]

nature research

Corresponding author(s): Marianna Kruithof-de Julio

Last updated by author(s): Dec 4, 2020

Reporting Summary

Nature Research wishes to improve the reproducibility of the work that we publish. This form provides structure for consistency and transparency in reporting. For further information on Nature Research policies, see our Editorial Policies and the Editorial Policy Checklist.

Statistics

For all statistical analyses, confirm that the following items are present in the figure legend, table legend, main text, or Methods section.

n/a	Confirmed	
☐	☒	The exact sample size (n) for each experimental group/condition, given as a discrete number and unit of measurement
☐	☒	A statement on whether measurements were taken from distinct samples or whether the same sample was measured repeatedly
☐	☒	The statistical test(s) used AND whether they are one- or two-sided<br>*Only common tests should be described solely by name; describe more complex techniques in the Methods section.*
☐	☒	A description of all covariates tested
☐	☒	A description of any assumptions or corrections, such as tests of normality and adjustment for multiple comparisons
☐	☒	A full description of the statistical parameters including central tendency (e.g. means) or other basic estimates (e.g. regression coefficient) AND variation (e.g. standard deviation) or associated estimates of uncertainty (e.g. confidence intervals)
☐	☒	For null hypothesis testing, the test statistic (e.g. F, t, r) with confidence intervals, effect sizes, degrees of freedom and P value noted<br>*Give P values as exact values whenever suitable.*
☐	☒	For Bayesian analysis, information on the choice of priors and Markov chain Monte Carlo settings
☐	☒	For hierarchical and complex designs, identification of the appropriate level for tests and full reporting of outcomes
☐	☒	Estimates of effect sizes (e.g. Cohen's d, Pearson's r), indicating how they were calculated

Our web collection on statistics for biologists contains articles on many of the points above.

Software and code

Policy information about availability of computer code

Data collection	Quantitative RT-PCR data was collected using the QuantStudio Design and analysis software (v 1.3.1). Flow cytometry data were collected using FACS Diva (v 8.0)

Data analysis

All software used was either open source or commercially available. For RNA-seq data, image analysis, base calling, and quality check was performed with the Illumina data analysis pipeline RTA3.4.4 and Bcl2fastq v2.20. Sequence reads were aligned using STAR (v2.7). Differential expression analysis was performed using the edgeR package (v3.30). Gene set enrichment analysis was performed using the R package fgsea (v1.12). For the analysis of transcriptomic signatures of the SPOP, FOXA1 and ETS genetic subgroups, raw-counts were retrieved using TCGABiolinks (v2.16) package in R statistical environment and normalization was performed using DESeq2 package (v1.26.0). For hierarchical clustering of LuCaP, BM18, LAPC9 and PNPCa PDXs, batch effect correction was performed using he empirical Bayes regression implemented in ComBat, in the R package sva (v3.35). For whole exome sequencing data for all PDX models, sequence alignment was performed to the GRC37/hg19 reference using Burrows-Wheeler Aligner (BWA, v0.7.12). Local realignment, duplicate removal and base quality adjustment were performed using the Genome Analysis Toolkit (GATK, v3.6). Somatic single nucleotide variants (SNVs) and small insertions and deletions (indels) were detected using MuTect (v1.1.4) and Strelka (v1.0.15), respectively, using the pool of normal as well as all mutations found in the non-TCGA subset of the Exome Aggregation Consortium database v0.3.1, and the Genome Aggregation Database (gnomAD v2.1.1, exome). To remove false positive mutations mouse-derived reads, we extracted human-derived reads with the package disambiguate (https://github.com/AstraZeneca-NGS/disambiguate, as of August 2020), followd by variant lookup with GATK HaplotypeCaller (v4.1.3.0). Allelespecific CNAs were identified using FACETS (v0.5.14). The segmented log2 ratio from WES was used as input for ABSOLUTE (v1.0.6). Clonality analysis was done with Pyclone (v.0.13.1). Clonal prevalence plot was done using the R package TimeScape (v.1.12.0). For Ion Torrent data, somatic mutation calling was performed using the PipeIT pipeline, which performs the initial variant calling step using Torrent Variant Caller (TVC, v5.0.3). Mutations called were normalised using bcftools (v1.10) and left aligned using GATK (v3.6). Statistical analyses were performed using GraphPad Prism (v.8.3), Flow cytometry data was analysed with FlowJo (v.10.6.2). Immunofluorescence images were processed and exported using ZEN (blue edition, v.2.5). Histological stainings were scanned using 3D Histech CaseViewer (v.2.3).

For manuscripts utilizing custom algorithms or software that are central to the research but not yet described in published literature, software must be made available to editors and reviewers. We strongly encourage code deposition in a community repository (e.g. GitHub). See the Nature Research guidelines for submitting code & software for further information.

Data

Policy information about availability of data

All manuscripts must include a data availability statement. This statement should provide the following information, where applicable:

- Accession codes, unique identifiers, or web links for publicly available datasets
- A list of figures that have associated raw data
- A description of any restrictions on data availability

Sequencing data have been deposited at the European Genome-phenome Archive (EGA), under accession number EGAS00001004673 and EGAS00001004675 and are publicly available.
RNASeq related to Sup.Fig.10D were deposited at the ENA (European Nucleotide Archive/Arrayexpress) under accession number E-MTAB-9656 and is publicly available

Field-specific reporting

Please select the one below that is the best fit for your research. If you are not sure, read the appropriate sections before making your selection.

[x] Life sciences   [ ] Behavioural & social sciences   [ ] Ecological, evolutionary & environmental sciences

For a reference copy of the document with all sections, see nature.com/documents/nr-reporting-summary-flat.pdf

Life sciences study design

All studies must disclose on these points even when the disclosure is negative.

Sample size

For the in vivo study, sample size was determined by performing two pilot experiments to assess expected mean difference between experimental groups. Variance and accuracy of the endpoint measures were determined, together with overall success rate of PDX engraftment and were used to calculate the minimum amount of observations required to reach sufficient power (N = 10 per group). The formula used was "E" = total amount of observation - total amount of groups. For the reported experiment, (10 x 2) observations - 2 experimental groups = 18. Acceptable range of E is commonly 10 to 20.
For near-patient studies, the sample size was determined basing on the recruitment capacity of the study as well as on biological material availability. Multiple independent measurements were taken for each patient-derived sample (drug screening, genetic sequencing, organoid formation assay). Sample size for the performed assays was optimized based on expected mean differences and according to assay manufacturer's indications (where applicable) and on previous experiments.

Data exclusions

No data had to be excluded. For the mutation analysis, several filters were applied in order to generate high-confidence variants. The filters applied are indicated in the Methods and were used on the basis of previous publications.

Replication

For all in vitro experiments, including RNA and whole-exome sequencing, biological over technical replicates were preferred and included whenever possible; a minimum of three technical replicates were collected for each experiment. For in vivo experiments, two pilot studies were performed prior to the in vivo study included in the manuscript and were used to determine sample size, optimal dose schedule and replicability. All attempts to replicate in vivo and in vitro studies were successful.

Randomization

Mice included in the in vivo experiment were randomized by body weight and tumor size after 7 days since PDX implantation and were divided into treatment and control groups (N=5 each). For ex vivo tumor slices assay, tumor slices were randomly assigned to the different treatment tested. For the in vitro organoid drug screening, replicates were distributed across different plates to account for assay variability.

	For all remaining in vitro assays, plate design was chosen to minimize manipulation time. Blanks and functional controls were consistently included for each in vitro experiment involving quantitative measurements, to reduce the effect of confounders and covariates.
Blinding	For the in vivo experiment, tumor growth assessment was done in single-blind mode. Due to the chemical nature of the compounds and their schedule of administration, blinding during treatment administration was not feasible. For molecular analyses, data collection and analysis were performed by different investigators, using arbitrary sample codes whenever possible, to minimize bias. Investigators were not blinded to group allocation during remaining in vitro data collection and analysis.

Reporting for specific materials, systems and methods

We require information from authors about some types of materials, experimental systems and methods used in many studies. Here, indicate whether each material, system or method listed is relevant to your study. If you are not sure if a list item applies to your research, read the appropriate section before selecting a response.

Materials & experimental systems

n/a	Involved in the study	
☐	☒	Antibodies
☒	☐	Eukaryotic cell lines
☒	☐	Palaeontology and archaeology
☐	☒	Animals and other organisms
☐	☒	Human research participants
☒	☐	Clinical data
☒	☐	Dual use research of concern

Methods

n/a	Involved in the study	
☒	☐	ChIP-seq
☐	☒	Flow cytometry
☒	☐	MRI-based neuroimaging

Antibodies

Antibodies used

CK5/6 Chemicon Milipore MAB1620; AR abcam ab133273; CK8 Thermo Fisher MA1-06318; Nkx3.1 AthenaES 314; p63 Abcam ab124762; p63 BD Pharmingen 559951; Ki67 Gene Tex GTX16667; CD44 BD Pharmingen 550988; panCK DAKO M0821; PD-L1 Cell signaling E1L3N clone, cat# 13684; PSA DAKO A0562; anti-mouse IgG A21202 AlexaFluor 488-labeled; anti-rabbit IgG A31572 AlexaFluor 555-labeled; CD4 Miltenyi Biotec 130-109-533 APC-Violet 770-labeled; CD127 Miltenyi Biotec 130-113-977 PE-Violet 770; CD25 Miltenyi Biotec 130-115-628 PE; CD3 BD Biosciences 564000 BUV 395; PD-1 BD Biosciences 367412 FITC; CD8a BD Biosciences 300924 PerCP-Cy 5.5; FoxP3 BD Biosciences 77-5776 APC; CD36 Miltenyi Biotec 130-110-879 PE-Violet 770; CD146 BD Biosciences 563619 AlexaFluor-647; PSMA Miltenyi Biotec 130-106-608 PE; E-Cadherin BD Biosciences 743715 Brilliant Violet 711; CD49f BD Biosciences 740416 Brilliant Violet 605.

Validation

The concentration and method specifications (e.g. antigen retrieval) were used based on the protocols of the antibody manufacturer`s. For IF applications, isotype controls matching the primary antibodies were used as negative controls.
Nkx3.1: pos.control prostate tissue, PMID: 17108105
p63 antibody validated on IHC and WB using Human epidermoid and breast carcinoma as pos.control. reacts with Human, Mouse, Rat
p63 validated on prostate tissue, https://doi.org/10.1073/pnas.0510652103
Ki67 validated on human cervical carcinoma and by Murai et al., PMID: 26965827.
CD44 human specific, validate by Kansas et al., PMID: 1702327
PSA validated IHC FFPE, reacts with human and mouse, tested on xenograft tissues (PMID: 25799167).
CK5/6: validated as per previous publications and reports available at the manufacturer's website. Positive control: human bladder tumor tissue, quality level MQ100.
AR: validated as per previous publications and reports available at the manufacturer's website. Positive control: human prostate.
CK8: validated by knockdown as per reports available at the manufacturer's website. Positive control used: frozen sections human colon.
panCK cytokeratins: validated as per previous publications and reports available at the manufacturer's website. The antibody shows an especially broad pattern of reactivity with human epithelial tissue.
PD-L1 validated as per previous publications and reports available at the manufacturer's website and as per routine testing at University of Bern Pathology department. Positive control used: human placenta tissue (shown in the manuscript).

All antibodies used for flowcytometry analyses were validated for this application by their respective manufacturers. In particular, the anti-FoxP3, anti-CD8a and the anti-PD-1 antibodies were lot-tested; the anti-E-cadherin, anti-CD44, anti-CD36, anti-PSMA and anti-CD49f antibodies were tested using the same fluorescent conjugate used in the reported experiments, the anti-CD146 and anti-CD3 antibodies were clone-tested. For the anti-CD4, anti-CD127 and anti-CD25 an extended validation is available on manufacturer's website. Validation was based on previous publications and on reports available at the manufacturer's website.

Animals and other organisms

Policy information about studies involving animals; ARRIVE guidelines recommended for reporting animal research

Laboratory animals

For PDX maintenance, NOD-scid IL2Rgammanull (NSG) and CB17 SCID male mice (6-week old) were used (Charles River France). For in vivo drug testing CB17 SCID male mice (6-week old) were used (Charles River France).

Wild animals	No wild animals were used in this study
Field-collected samples	No field-collected samples were used in this study
Ethics oversight	Cantonal Veterinary Ethical Committe, Switzerland (BE55/16)

Note that full information on the approval of the study protocol must also be provided in the manuscript.

Human research participants

Policy information about [studies involving human research participants]

Population characteristics	All included research participants were male, we included detailed demographic characteristics in SI Table 6.
Recruitment	Participants who underwent radical prostatectomy (primary) or recurrence resections were recruited in this study after providing written informed consent. Selection bias was limited as much as possible, however the study population was recruited on a single-center basis, enriching for a population of caucasian men. Additional demographic variables were reported in Supplementary Data 6 (SI Table 6)
Ethics oversight	Cantonal Ethical Committe, Switzerland (KEK 06/03 &2017/02295)

Note that full information on the approval of the study protocol must also be provided in the manuscript.

Flow Cytometry

Plots

Confirm that:

- [x] The axis labels state the marker and fluorochrome used (e.g. CD4-FITC).
- [x] The axis scales are clearly visible. Include numbers along axes only for bottom left plot of group (a 'group' is an analysis of identical markers).
- [x] All plots are contour plots with outliers or pseudocolor plots.
- [x] A numerical value for number of cells or percentage (with statistics) is provided.

Methodology

Sample preparation	For all assays, cells were washed twice in FACS wash before staining. When antibody staining was performed, human Fc receptors were blocked using human FcR Blocking Reagent at 1:20 dilution in FACS wash for 10 minutes and then directly stained with the appropriate antibody mix for 20 minutes at room temperature. CD3-enriched cells used for MLR assays were stained with CellTrace Violet 5uM at the beginning of the experiment. For Treg assay, cells were further stained for FoxP3 using the FoxP3 staining Buffer Set (eBioscience) according to manufacturer's instructions. For PNPCa cell staining, cells were incubated in DAPI to discriminate live/dead cells. Compensation controls were acquired independently for every experiment and were generated using AbC Total Antibody compensation beads kit (Thermofisher Scientific) following manufacturer's instructions.
Instrument	All experiments were acquired using an LSR-II instrument (BD Biosciences)
Software	Flow cytometry data were analysed with FlowJo (v.10.5)
Cell population abundance	A minumum of 50.000 events were acquired for each experimental sample. For compensation controls, 10.000 events were acquired.
Gating strategy	Gating strategy is reported in Supp. Fig. 18B-D for MLR assay, Treg assay and PNPCa cells staining respectively.

- [x] Tick this box to confirm that a figure exemplifying the gating strategy is provided in the Supplementary Information.
